# Near extinction of the HBV quasispecies driven by the hard selective sweep in chronic hepatitis B

**DOI:** 10.1128/mbio.01113-25

**Published:** 2025-06-30

**Authors:** Daiqiang Lu, Andong He, Guichan Liao, Renyu Zhou, Zichun Cheng, Ka Cheuk Yip, Xiufang Wang, Wei Cao, Jiaojiao Peng, Ruiman Li, Jie Peng, Feng Gao

**Affiliations:** 1 Institute of Molecular and Medical Virology, School of Medicine, Jinan University 470005, Guangzhou, China; 2 Department of Obstetrics and Gynecology, The First Affiliated Hospital of Jinan University 162698, Guangzhou, China; 3 Department of Infectious Diseases, Nanfang Hospital, Southern Medical University 658729 , Guangzhou, China; 4 Key Laboratory of Viral Pathogenesis and Infection Prevention and Control, School of Medicine, Jinan University 470005, Guangzhou, China; University of Ljubljana, Ljubljana, Slovenia

**Keywords:** hepatitis B virus, evolution, selection, escape, diversity

## Abstract

The viral quasispecies play an important role in pathogenesis. However, little is known about the nature of hepatitis B virus (HBV) quasispecies and its impact on the outcomes of chronic hepatitis B. Here, we characterized the bona fide quasispecies viral populations from 16 chronically infected patients by analyzing the near full-length viral genomes obtained by single genome sequencing. Follow-up plasma samples were collected from six patients after 6 months and from four patients after 3–5 years. We found that the viral population diversity levels were highly variable among these patients, depending on the status of the selection pressure. To further understand the host selection pressure over time, we compared viral sequences from different timepoints in 10 patients and found the hard selective sweep (HSS) at the whole genome level in three of them. This HSS was extremely strong and drove the highly diversified viral quasispecies into a distinct yet homogeneous viral population in each patient after 1–4 years of infection. The identical or nearly identical sequences in the homogeneous viral population indicate that they originate from a single infected source. Predominant and fixed mutations were observed in well-characterized T cell epitopes but not in known epitopes of neutralizing antibodies or N-linked glycosylation sites. Since HSS is strong enough to drive HBV quasispecies into near extinction and frequently occurs in chronic hepatitis B patients, this raises the hope for eliminating HBV from the last single reservoir through enhancing specific T cell immune responses targeting the highly selected homogeneous viruses.

**IMPORTANCE:**

The nature of bona fide hepatitis B virus (HBV) quasispecies and its potential impact on the outcomes of chronic hepatitis B are not well understood. Analysis of the high-quality near full-length HBV sequences from chronic hepatitis B patients identifies the strong hard selective sweep that can drive the viral quasispecies into a distinct yet highly homogeneous viral population. The nearly identical viral population suggests that all these viruses are from a single source. Predominant mutations were identified in known T cell epitopes but not in epitopes for neutralizing antibodies, indicating that the hard selective sweep is most likely driven by T cell selection pressure. These findings indicate that enhancement of specific T cell responses during chronic hepatitis B can easily eliminate the highly homogeneous viral population and serve as a promising approach to achieve the goal of a functional cure for chronic hepatitis B.

## INTRODUCTION

Hepatitis B is an infection of the liver caused by the hepatitis B virus (HBV). Although HBV is cleared by immune responses during acute infection in the majority of the infected people, ~5% of them become chronic hepatitis B (CHB), which can lead to hepatocellular carcinoma (HCC) (1–3). About 254 million people were living with CHB, resulting in ~1.1 million deaths in 2022 (4). Each year, 1.2 million new infections are diagnosed. Thus, hepatitis B is a major threat to public health (4). HBV is highly variable due to the error-prone reverse transcriptase and can result in a diversified viral population, termed quasispecies, in a CHB patient (5, 6). Previous studies showed that the viral quasispecies in a host plays an important role in the pathogenesis of polioviruses and hepatitis C viruses (HCV) (7, 8). The HBV quasispecies can also affect viral evolution, pathogenesis, drug discovery, and vaccine design. For example, mutations in the *X* gene are closely associated with the development of HCC (9). Furthermore, HCC patients exhibit a higher frequency of *PreS* gene deletions along with increased complexity and diversity of viral populations (10, 11). Additionally, immune selection pressure-driven enrichment of *PreCore* and/or basal *Core* promoter mutants contributes to serological conversion in HBeAg-positive patients (12). Furthermore, genetic variations in the reverse transcriptase gene and the major hydrophilic region (MHR) lead to the emergence of drug-resistance mutations and neutralizing antibody (nAb) escape mutations, respectively (13–16). These mutations have important impacts on drug discovery and multivalent vaccine design.

However, HBV quasispecies have not been well characterized due to the limitations of the used methods. Direct sequencing of bulk PCR products can only detect the consensus sequences of the quasispecies population in the PCR products but not the actual individual sequences (17, 18). Cloning analysis of bulk PCR products can detect individual sequences but suffers greatly from resampling of the amplified same genome (19) and Taq-polymerase introduced mutations during PCR (17). Next-generation sequencing has very short read lengths (50–300 bp) and higher error rates (0.1%) (20). These above methods also employ the bulk PCR amplification step and can be significantly affected by the highly artificial recombination rate during bulk PCR amplification (21–23). HBV sequences have been obtained using the above methods, and only one recent study used the limiting dilution PCR method to analyze the drug resistance to lamivudine after treatment failure with antiviral drugs (24).

The single genome sequencing (SGS) method has been used extensively to study HIV, HCV, and malaria quasispecies (25–27). We have used the SGS method to study HIV quasispecies and made numerous important discoveries. They include accurate determination of transmitted/founder HIV-1 strains (18), the co-evolution of the viruses and broadly neutralizing antibodies (bnAbs) in the same host (25), and mechanisms of immune escape from bnAbs (28). Since PCR is carried out with a single genome and the final PCR products are sequenced in bulk, SGS is not affected by Taq-polymerase-mediated mutations, resampling, and artificial recombination as the above-mentioned methods (17–23). Therefore, SGS provides an excellent method for the unbiased analysis of viral quasispecies and the accurate capture of the *in vivo* evolutionary trajectory of HBV in CHB patients.

A selective sweep often occurs under host selection pressure during viral infections (29, 30). In the soft selective sweep (SSS), beneficial mutations are present on different genomic backgrounds, and it does not eliminate all genetic variation in the population (31). However, in the hard selective sweep (HSS), a beneficial mutation increases in frequency rapidly and drastically reduces genetic variation in the population (29, 30). SSS is often observed in HIV, HCV, and SARS-CoV-2 infections as well as during influenza A virus transmission (32–35). However, HSS has been found during HIV and HCV transmission (18, 36) or after treatment failure of HIV infection (37–39), but it has not been observed during natural chronic infection of any viruses. About 1% of the CHB patients each year can become HBsAg negative and free of HBV infection (40–42). The virologic attributes to this self-clearance of HBsAg have not been investigated. It will be important to investigate whether HSS plays a critical role in the HBsAg self-clearance during natural HBV infection.

Here, we analyzed the near full-length (NFL) HBV genome sequences of longitudinal samples from the same CHB patients using the SGS method, which can precisely characterize the viral quasispecies without interference of the error rate, resampling, and artificial recombination generated by bulk PCR (17–22). We found that the strong HSS that resulted in a highly homogeneous viral population often occurred during natural CHB. This unique phenomenon demonstrates the presence of the previously unappreciated host selection pressure that is strong enough to eliminate all diverse viral strains except one homogeneous viral population. This finding has important implications in understanding the biology, reservoir, and pathogenesis of HBV as well as developing effective treatment strategies for CHB.

## RESULTS

### Genetic diversity levels of HBV are highly variable in CHB patients

**Fig 1:**
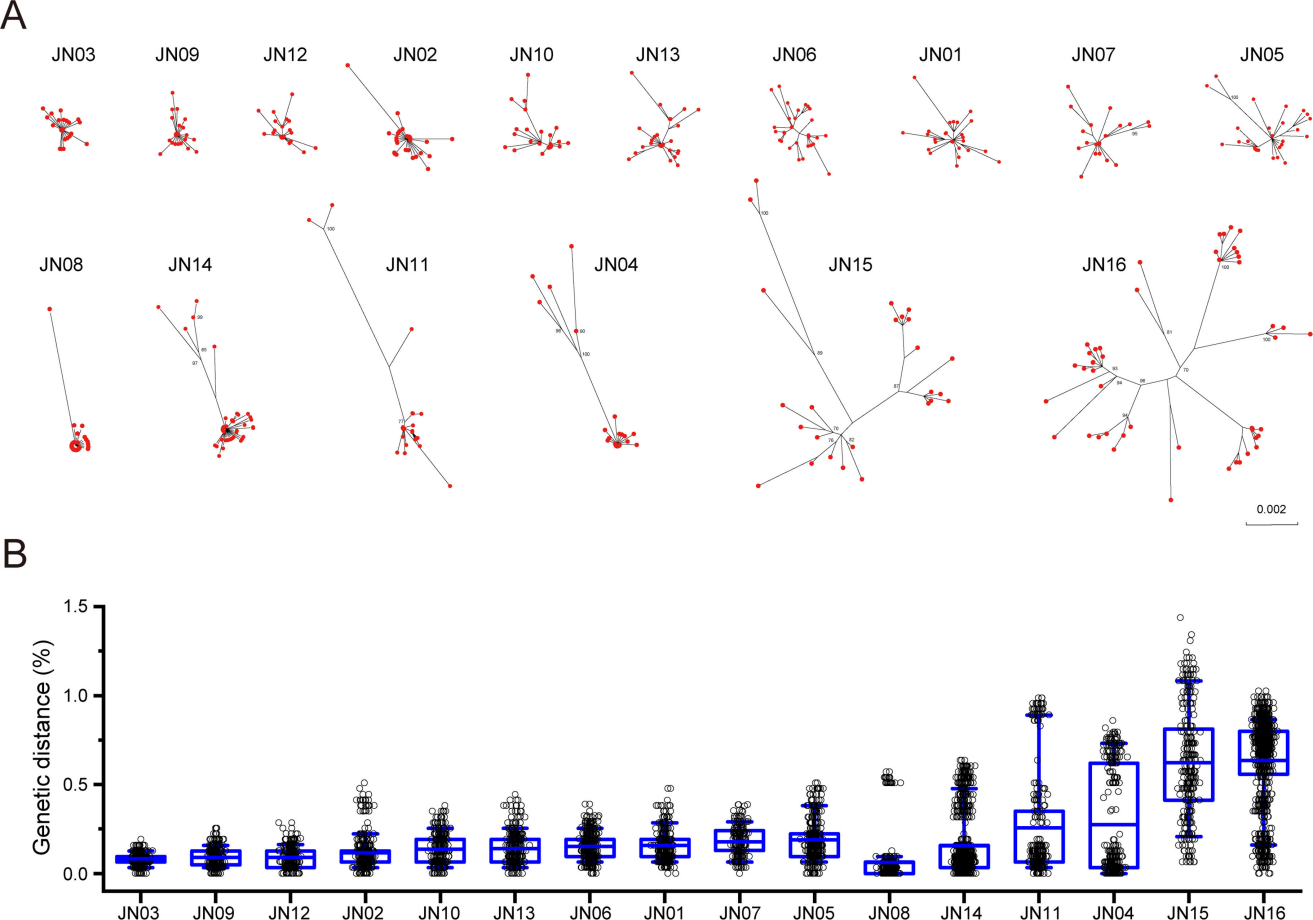
Various levels of genetic diversity among the chronically HBV-infected patients. (**A**) Phylogenetic tree analysis of all NFL HBV sequences from each patient was constructed by the maximum likelihood method using the General Time Reversible Model (GTR) model. The branch reliability was estimated with 1,000 bootstrap replicates. (**B**) The intra-host genetic diversity levels were compared among 16 CHB patients. The genetic diversity within a patient was determined by calculating pairwise genetic *P*-distances of the HBV sequences from the same patient. The blue middle line indicates the mean. The box shows 25–75 percentiles of the diversity, and the whiskers show 10–90 percentiles of the diversity.

The HBV quasispecies in the samples collected from 16 CHB patients were studied (Table S1). All but three were infected for >10 years. The average of viral loads was 4.51 × 10^7^ (9.41 × 10^2^ to 5.45 × 10^8^) IU/mL. To accurately characterize the viral population, we obtained an average of 29 (20–57) NFL viral genome sequences from each archived plasma sample by SGS (Fig. 1; Table S1). Phylogenetic analysis showed that sequences from each patient formed a tight lineage (Fig. S1). The HBV genetic diversity levels were highly variable among these patients. It was less than 0.2% (0.08%–0.19%) in 10 patients (Fig. 1; Table S2). In the other four patients (JN08, JN14, JN11, and JN04), the genetic diversity levels of the major viral populations were even less than 0.1% (0.03–0.09) but each contained a highly divergent viral population (Fig. 1A; Fig. S2). The last two patients (JN15 and JN16) had much higher genetic diversity levels and harbored multiple lineages of divergent variants (Fig. 1; Fig. S2). The highly variable genetic diversity levels indicate that the viruses are under different selection pressures among CHB patients.

### HSS drives the diverse viral quasispecies into a highly homogeneous viral population

**Fig 2:**
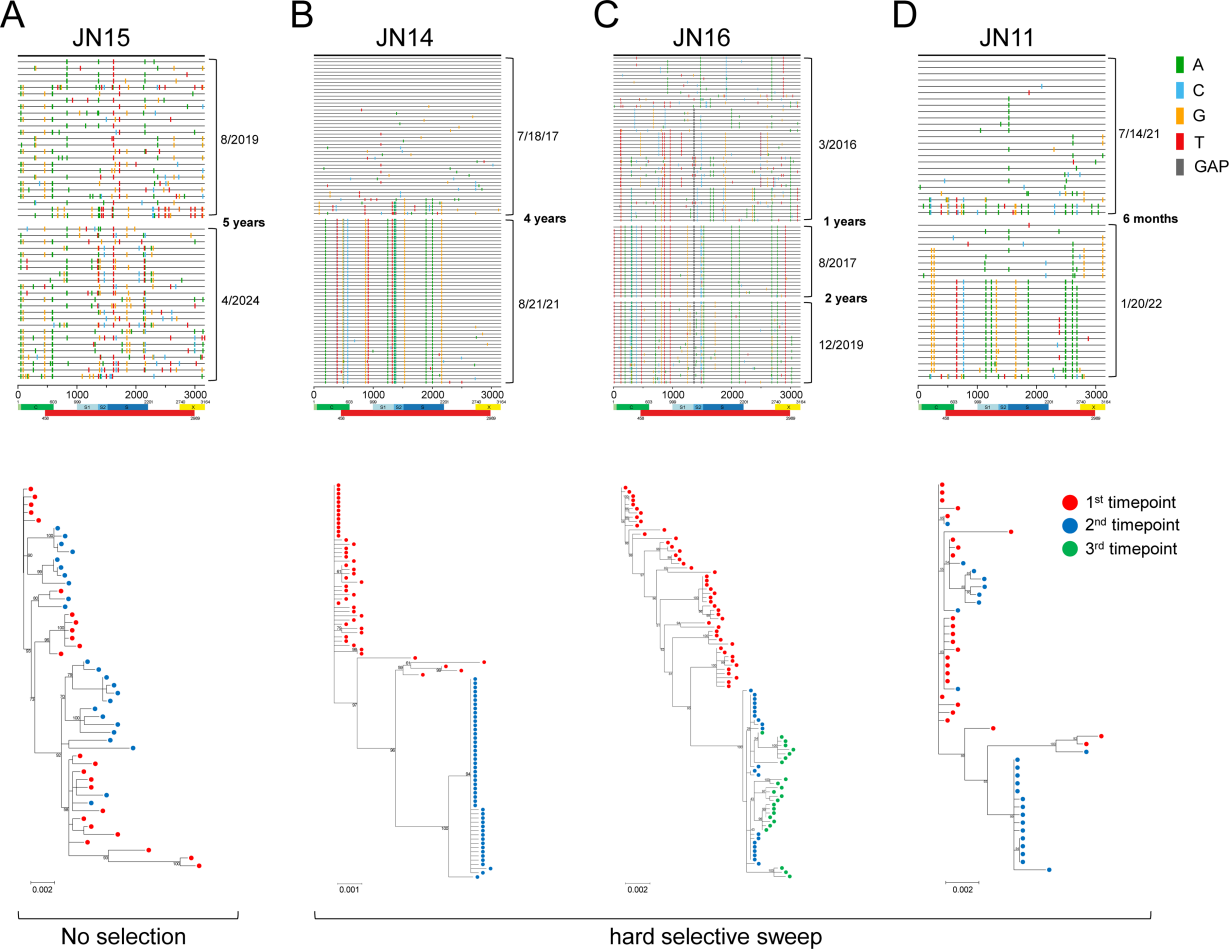
HSS in the chronically HBV-infected patients. The multiple NFL HBV genome sequences obtained by SGS at 2–3 timepoints from each CHB patient were analyzed. Each highlighter plot shows the positions of all mutations in each HBV genome sequence, compared to the consensus sequence of all sequences obtained at the first timepoint (top thick line). Each line represents an NFL HBV genome sequence. Mutations and gaps are color coded, and their locations in the viral genome are shown at the bottom. The phylogenetic trees were constructed by the maximum likelihood method with the GTR model. Its reliability was estimated by 1,000 bootstrap replicates. The colors of the dots represent viral sequences from different timepoints. The viral population in patient JN15 was under no selection (**A**), while the viral populations in patients JN14 (**B**), JN16 (C), and JN11 (**D**) were under HSS.

To understand what caused the high levels of viral genetic variation and how HBV evolves over time, we analyzed the viral quasispecies from additional timepoints (0.5–5 years later) from 10 CHB patients (Table S1). In seven cases (70%), the viral sequences were indistinguishable between the initial timepoint and the second timepoint (Fig. 2A; Fig. S3), similar to most results in previous reports (43, 44). This showed that the viral populations in these CHB patients were not likely under detectable immune selection pressure, consistent with the reported immunity exhaustion during CHB (45–49). However, all viral sequences in JN14 at the second timepoint were distinct from those at the first timepoint and formed an independent monophyletic branch (Fig. 2B). Importantly, they were highly homogeneous, with the majority (64.6%) of the NFL genome sequences identical to each other, while all but two other sequences differed from the majority sequence by only one nucleotide. This demonstrates that the viruses experienced a strong HSS.

**Fig 3:**
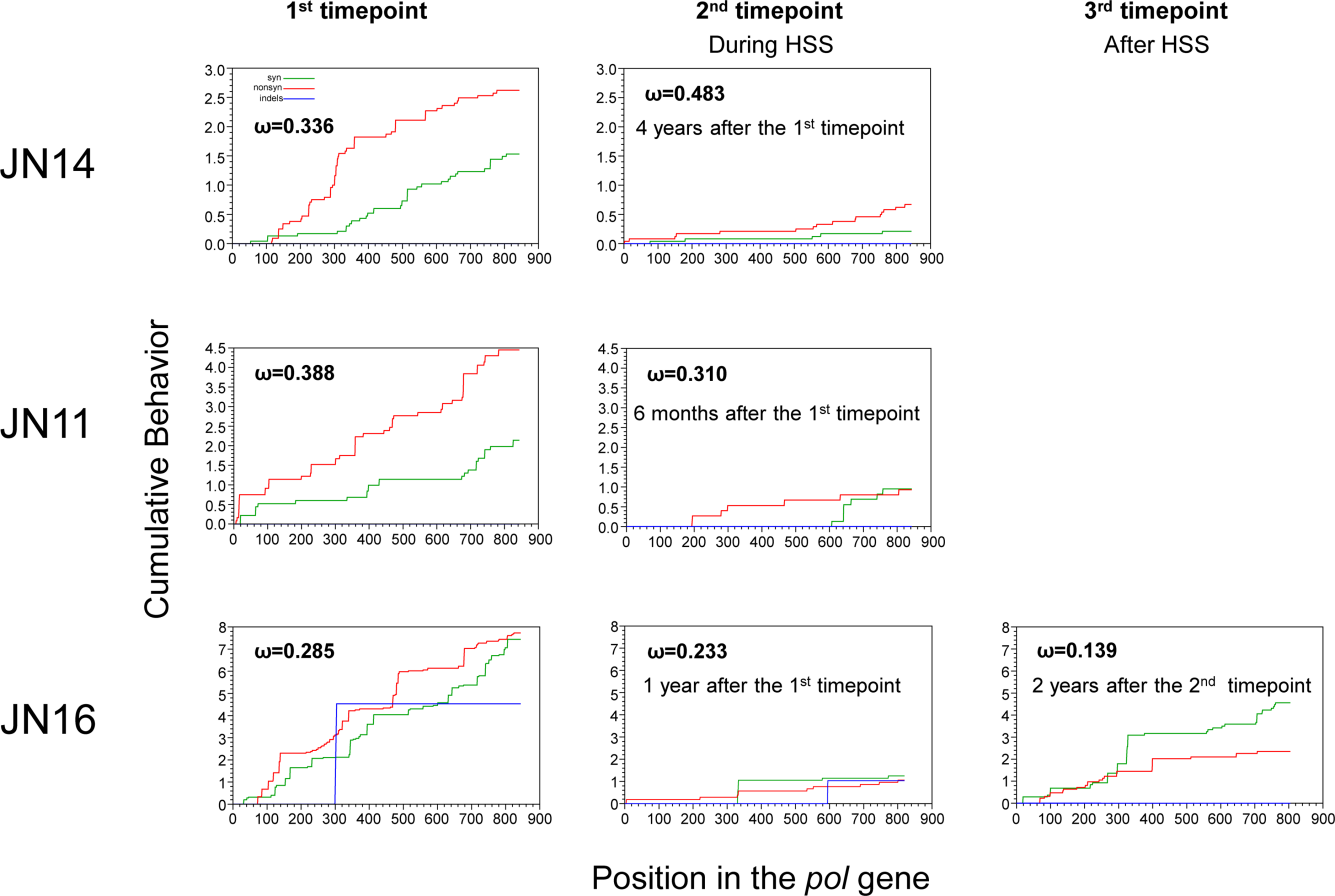
Severe reduction of both synonymous and nonsynonymous mutations during HSS. Cumulative plots of each codon average behavior for all sequences compared for the HBV *Pol* gene at different timepoints for synonymous mutations (green), nonsynonymous (red) mutations, and indels (blue). Values of ω denote average ratios of the rate of nonsynonymous substitutions per nonsynonymous site (dN/dS) for each sample.

A similar strong HSS on the viruses was observed in JN16. The viral population at the second timepoint was also very different from those from 1 year ago and formed a monophyletic branch (Fig. 2C). The majority of sequences (76.2%) were highly homogeneous (identical to each other or different by two nucleotides) after HSS, while the other five sequences differed from the majority sequences by only three nucleotides. We also obtained a third timepoint sample (2 years after the second timepoint) from the same patient. Interestingly, all the viral sequences at the third timepoint evolved from the viruses at the second time (Fig. 2C). This demonstrates that the highly homogeneous viral population selected by the strong HSS can continue to evolve afterward. No detection of any viral sequences from the first timepoint at the third timepoint indicates that the diverse viral variants at the first timepoint were eliminated through HSS.

In the third patient JN11, the second timepoint sample was collected only 6 months later. The majority of the sequences (60%) at the second timepoint were also highly homogeneous and distinct from those at the first timepoints (Fig. 2D), like in JN14 and JN16. All (15) but one sequence was either identical or differed from the majority of sequences by only one nucleotide. However, nine sequences (36%) were still indistinguishable from those at the first timepoint (Fig. 2D). These results indicate that the viral quasispecies were in the process of being replaced by the highly homogeneous viral population under the strong HSS.

### Recombination facilitates HSS

In patient JN14, four sequences from the first timepoint were divergent but more closely related to the sequences from the second timepoint (Fig. S4A). The highlight plot showed that eight bases in the *S* gene were shared between these four sequences and those from the second timepoint after HSS. This indicates that the two halves of the genomes had different origins. Analysis of the 5’-half sequences (~1,300 bp) showed that all the sequences at the second timepoint were distinct from those from the first timepoint (Fig. S4B). However, the analysis of the 3’-half sequences (~1,800 bp) showed that all the new mutations in the sequences from the second timepoint were present in those four divergent sequences at the first timepoint (Fig. S4C). Phylogenetic tree analysis showed that all the 5’-half genome sequences from the second timepoint formed an independent lineage, while the 3’-half genome sequences formed an independent tight cluster together with those four divergent viruses from the first timepoint. These results strongly indicate that the recombination occurred between a minority population of the variants and an undetected viral population at the first timepoint and only the recombinant virus survived the strong HSS (30).

### Limited mutations in the viral population due to the strong selective pressure

After HSS, both synonymous and nonsynonymous mutations across the entire *Pol* gene, which consist of the majority (80%) of the viral genome, were severely reduced in all three individuals (Fig. 3), consistent with the highlighter plot analysis results (Fig. 2). Similar results were also observed for the other three genes (*PreC_Core*, *PreS1_S2_S*, and *X*) which completely or partially overlap the *Pol* gene (Fig. S5). However, both synonymous and nonsynonymous mutations in the viral genomes that did not undergo HSS showed similar rates between two timepoints (Fig. S6). Interestingly, after the new homogeneous viral population completely replaced the previous viral quasispecies due to HSS in JN16, the numbers of synonymous and nonsynonymous mutations started to increase (Fig. 3), indicating that the host selection pressure exerted by HSS was no longer able to suppress these newly selected homogeneous viruses, and the viruses continued to evolve (Fig. 3 and 2C).

### Identification of predominant/fixed mutations in known T cell epitopes after HSS

After HSS, a number of mutations were fixed (100%) or predominant (>90%) in the viral population in each patient, indicating that they were strongly selected. These mutations together caused a dramatic viral population shift in all three HSS patients. To investigate whether these mutations were under selection pressure from immune responses, we determined whether any of these mutations occurred in known T and B cell epitopes. The MHR of HBsAg (aa 99–169) containing the “a” determinant is the main target for nAb (50). Examination of the amino acid substitutions in MHR did not show any strongly selected predominant/fixed mutations before and after HSS among all three patients (Fig. S7). In addition, no amino acid substitutions resulted in changes at N-linked glycosylation (NLG) sites in the entire S protein. Thus, nAbs did not likely play a role during HSS in these CHB patients.

**Fig 4:**
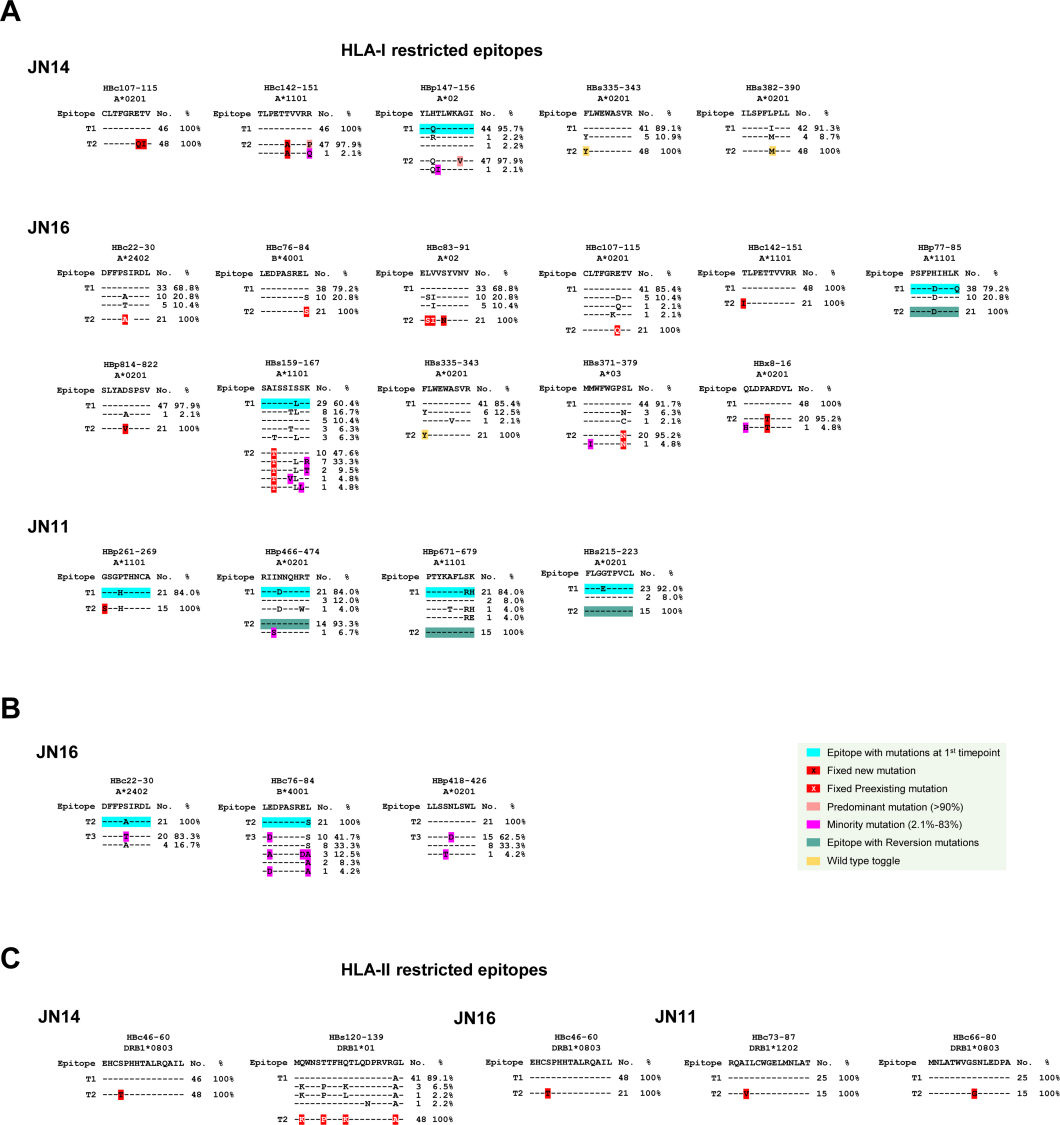
Identification of predominant and fixed mutations in well-characterized T cell epitopes. (**A**) The sequence alignments of the well-characterized T cell epitopes restricted by HLA-I with viral sequences with predominant/fixed mutations from JN14, JN11, and JN16 before and after HSS. (**B**) The sequence alignments of the well-characterized T cell epitopes restricted by HLA-I with viral sequences with mutations between the HSS-selected viruses and the newly evolved viruses in JN16. (**C**) The sequence alignments of the well-characterized T cell epitopes restricted by HLA-II with viral sequences with predominant/fixed mutations from JN14, JN11, and JN16 before and after HSS. Various kinds of mutations are indicated by different color schemes as shown in the legends.

The HBV-specific T cell responses are extremely weak, which has made it very difficult to analyze in CHB patients (51, 52). We also only had plasma samples from the patients, which prevented us from performing T cell immune response analysis. However, we were able to determine whether any of these predominant/fixed mutations selected by HSS were present in well-characterized T cell epitopes and thus potentially escaped from T cell responses. In JN14, 17 mutations were strongly selected by HSS. They were either predominant (>97%) or fixed in the viral population after HSS (Fig. S7A). Seven mutations were found in five known HLA-I-restricted epitopes (Fig. 4A; Table 1). The two other mutations (F335Y and I387M) in the S protein were known wild type (53). Therefore, they were not likely under the pressure of T cell selection.

**TABLE 1:** Number of predominant and fixed mutations in known T cell epitopes

	Total mutations	No. of nonsynonymous mutations in known T cell epitopes					No. of nonsynonymous mutations not in known epitopes	No. of synonymous mutations
		HLA-I restricted			HLA-II restricted			
		1	2	3	1	3		
JN14	17	HBp147-156 HBs335-342 HBs382-390	HBc107-115 HBc142-151		HBc46-60	HBs120-139	5	1
JN16	21	HBc22-30 HBc76-84 HBc107-115 HBc142-151 HBp77-85 HBp814-822 HBs159-167 HBs335-343 HBs371-379 HBx8-16		HBc83-91	HBc46-60		5	3
JN11	13	HBp261-269 HBp466-474 HBs215-223	HBp671-679		HBc73-87 HBc66-80		3	3

Among 21 predominant/fixed mutations identified in JN16, 12 were found in 11 known HLA-I-restricted epitopes (Table 1). One preexisting mutation F335Y in the HBs335-343 epitope of the S protein was known as wild type as seen in JN14. One mutation in a *Pol* epitope (HBp77-85) was a reversion mutation that mutated back to the wild type. Thus, these two mutations should not be associated with escape from T cell responses. Interestingly, from the second timepoint (highly homogeneous viral population selected by the strong HSS) to the third timepoint, the viruses continued to evolve and became more diversified (Fig. 2C and 3). Three new predominant mutations (67%–83%) were detected in three known HLA-I-restricted epitopes (Fig. 4B; Fig. S7B). In addition, two or three different amino acid substitutions were found in two epitopes. These indicate that the viruses were under a new round of selection from the T cell immune responses after HSS. However, no fixed escape mutations and multiple different mutations in the same T cell epitopes indicate that it was still at the early stage of the T cell selection.

There were 13 fixed mutations in JN11. Five of them were found in four known HLA-I-restricted epitopes (Fig. 4A; Fig. S7A). However, only one in a *Pol* epitope (HBp261-269) was a potential escape mutation, while all the other three are reversion mutations. Interestingly, all four epitopes already had mutations at the first timepoint, suggesting that they all had escaped from the earlier T cell selection pressure.

The fixed mutations were also found in one or two known HLA-II-restricted epitopes in each patient (Fig. 4C; Table 1). In addition, many mutations were not found in known T and B cell epitopes in each patient (Fig. S7; Table 1). Their roles warrant more in-depth analysis in future studies. Taken together, the identification of the potential escape mutations in well-characterized T cell epitopes indicates that escape from T cell immune response plays an important role in HSS.

### HSS increases the evolutionary rate of HBV

**Fig 5:**
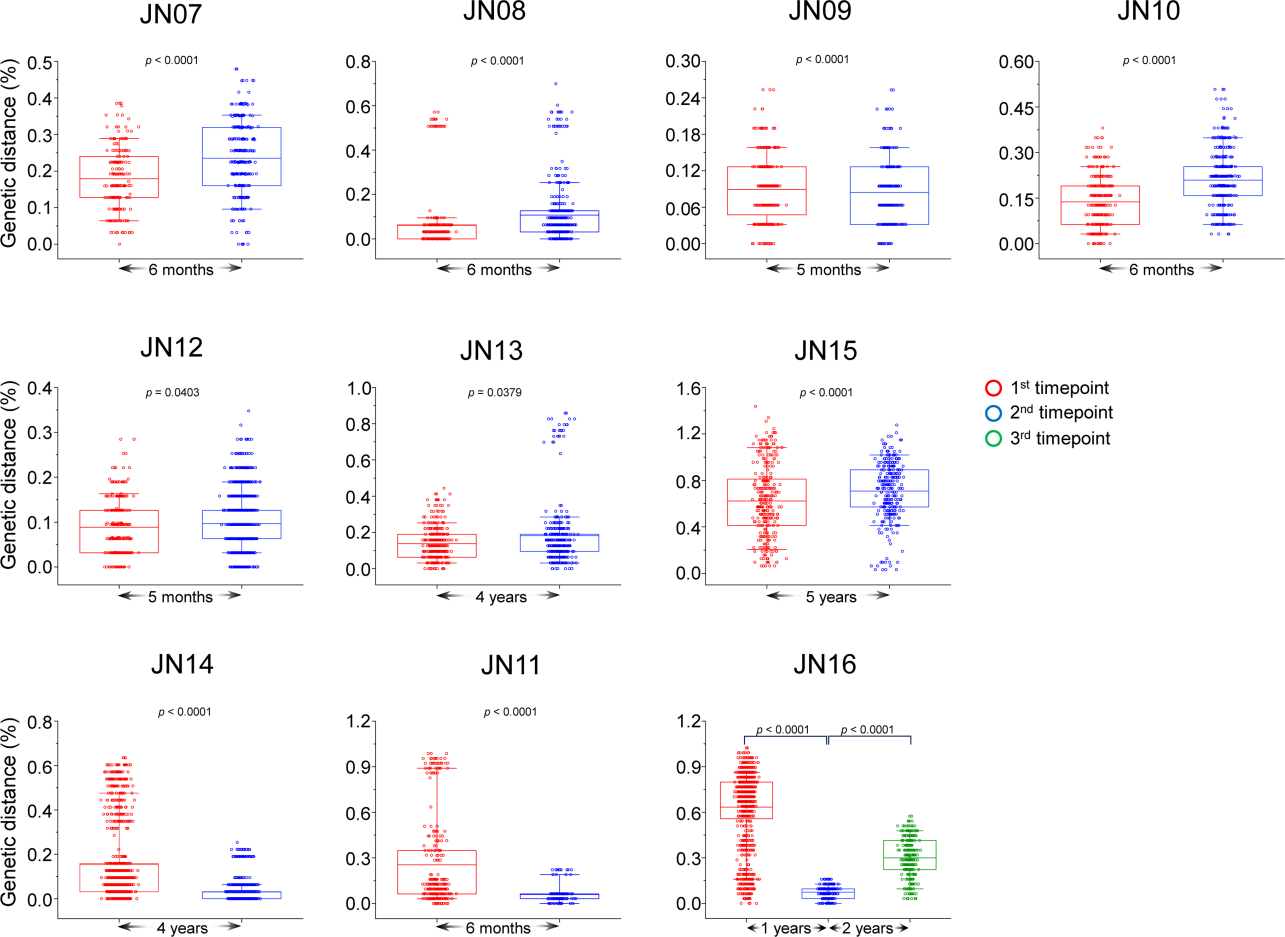
Strong reduction of genetic diversity levels after the HSS. The genetic diversity was determined by calculating the *P*-distance of viral sequences from each timepoint sample. The pairwise genetic distances from different timepoints in each patient were compared. The first, second, and third timepoint samples were indicated by red, blue, and green circles, respectively. Statistical comparisons were carried out using the Mann-Whitney *U* test or Kruskal-Wallis test.

Analysis of the sequences from two different timepoints from each patient showed increases in the average diversity levels of the viral population in the CHB patients without HSS over time (Fig. 5). This indicates that the viruses continuously evolved, although the viruses from the two different timepoints were not fully distinguishable from each other (Fig. 2A; Fig. S3). However, the diversity levels of the viral populations were significantly decreased during HSS in all three CHB patients (Fig. 5). The Shannon entropy analysis of the sequences at two different timepoints for each patient also showed the same result. The Shannon entropy of the viral population after HSS was dramatically reduced in the three CHB patients, while it was maintained at similar levels at both timepoints in the CHB patients without HSS (Fig. S8). With these sequences from longitudinal samples, we were able to compare the HBV evolutionary rate in patients with or without HSS (Table 2; Table S3). The average evolutionary rate was 2.63 × 10^−4^ substitutions per site per year for viruses without HSS, consistent with the previous reports (3.7 × 10^−6^ to 7.72 × 10^−4^) (43, 54–59). However, it was 4.77 × 10^−3^ substitutions per site per year for viruses with HSS (Table 2). It was as high as 1.14 × 10^−2^ for JN16. Thus, the evolutionary rate of HBV under HSS is about 10–100 times faster than HBV not under HSS (Table 2; Table S3). Interestingly, the evolutionary rate of HBV returned to the normal rate (8.56 × 10^−4^ per site per year) after HSS in the same patient JN16. This demonstrates that the HBV evolutionary rate can dramatically increase during HSS.

**TABLE 2:** Fast HBV evolution rate during the HSS^*a*^

PID	Evolutionary rate	Selection
JN07	1.43 × 10^−4^	No HSS selection
JN08	6.30 × 10^−5^	
JN09	4.78 × 10^−5^	
JN10	8.02 × 10^−4^	
JN12	2.58 × 10^−4^	
JN13	6.97 × 10^−5^	
JN15	4.58 × 10^−4^	
JN14	1.09 × 10^−3^	HSS selection
JN16	1.14 × 10^−2^	
JN11	1.81 × 10^−3^	

^*a*^ Evolutionary rates are expressed as nucleotide substitution per site per year.

## DISCUSSION

The strong HSS on the whole viral genome has been observed during transmission and under selection of antiviral drugs in HIV and HCV (18, 36–39). However, such a strong HSS on viral populations has not been observed within a host during chronic viral infection. By analyzing the bona fide quasispecies viral populations from the longitudinal samples, we found that strong HSS frequently occurred among CHB patients (30%), and it was so strong that it drove the viral quasispecies into a highly homogeneous viral population. The nearly identical NFL sequences in the viral population after HSS strongly suggest that this homogeneous viral population originated from a single infected cell, while HBV in all but one infected cell was eliminated during HSS. None of the initial diverse viruses were detected more than 2 years after the HSS in one patient. This further confirms that the diverse viruses have been selected out by HSS.

No mutations were found in the HBsAg MHR region, which is the only known target for nAbs, and all three CHB patients were not treated with antiviral drugs (Table S1). Thus, this strong HSS was not likely caused by nAbs or antiviral drugs. However, the predominant/fixed mutations were detected in the well-characterized T cell epitopes in all three patients, suggesting that T cell immune responses play an important role in HSS. In addition, some fixed mutations were also found at sites not associated with known T cell epitopes. These mutations might also play a role in the strong HSS if they are present in T cell epitopes that have not been identified yet. Thus, it will be important to characterize these predominant/fixed mutations in or outside known T cell epitopes to investigate their roles in escape from T cell immune responses during HSS in future studies.

About 95% of acute HBV infections can be cleared, demonstrating the important role of the host immune system (1). Even though both B and T cell immune responses are generally exhausted during CHB (45–49), the self-clearance of HBsAg still naturally occurs in about 1% of HBsAg-positive patients per year (40–42). This indicates the host immune system still can eliminate the viruses during CHB in rare cases, but the mechanism of HBsAg self-clearance remains unresolved. In this study, we showed that the selection force of HSS was so strong that it drove the diverse viral population into a highly homogeneous viral population, possibly from a single infected cell, in CHB. This indicates that the immune responses, likely the T cell responses, can drive HBV very close to extinction during chronic infection. If the selection pressure that causes HSS can be boosted a little more to eliminate the highly homogeneous viruses from the last remaining single HBV-infected cell, this can lead to a functional cure of CHB as seen in the self-clearance of HBsAg (40, 60).

Our analysis of the JN14 sequences showed that the viruses at the second timepoint were like the result of recombination. One recombinant fragment contained eight mutations that were detected in the minority viral population (four sequences) at the first timepoint, while the other recombinant fragment contained nine new mutations that were not detected in the viral population at the first timepoint. Thus, it is very likely that these viruses were generated through recombination between those divergent minority viruses and an undetected virus population. Such recombinants have been frequently observed during HIV-1 evolution (19, 21, 61). Importantly, these results indicate that recombination may be an important way for viruses to survive the strong HSS.

Because of the dramatic viral population change, the evolutionary rate of HBV increased by 10- to 100-fold during HSS. Thus, HBV does not evolve linearly in the infected hosts over time. Thus, the genetic diversity levels of the viral populations will depend on the time when the samples are collected. It will be highly diverse in CHB patients without HSS but highly homogeneous in CHB patients right after HSS, no matter how long the infections are. Importantly, the much faster evolutionary rate caused by HSS also has a significant impact on the estimation of the molecular evolutionary rate of HBV.

Overall, our results show that the frequent occurrence of HSS in CHB patients significantly impacts HBV evolution, the diversity of the viral population, and the pathogenesis of CHB. HSS is so forceful that only one infected cell is not eliminated in the CHB patient, very close to the extinction of HBV. Viral load assays have been frequently used to monitor the antiviral treatment efficacy in CHB patients. Thus, the blood samples are readily available and can be easily used for the SGS analysis to determine if the viruses in the patients experience HSS. Our data show that the viruses under HSS can last for about 1 year. This gives sufficient time to monitor the presence of HSS during CHB. Since T cell immune responses are most likely the forces that cause HSS, closely monitoring viral population changes, identifying T cell immune responses leading to HSS, and enhancing the T cell responses can serve as a promising approach to cure CHB by eliminating the highly homogeneous viral population or the last infected cell during HSS.

## MATERIALS AND METHODS

### Patients

Sixteen CHB patients were enrolled in this study. Twelve CHB patients (JN01–JN12) were admitted to the First Affiliated Hospital of Jinan University. Among them, follow-up samples were collected from six patients 6 months later (JN07–JN12). Four CHB patients (JN13–JN16) were admitted to Nanfang Hospital of Southern Medical University. Written consent was obtained from all patients, and the study was approved by the ethics committee of The First Affiliated Hospital (KY-2022-044) and Nanfang Hospital of Southern Medical University (NFEC-2020-290). One follow-up sample was collected from each patient 3–5 years later from three patients (JN13–JN15). Two samples from two timepoints (1.4 and 3.75 years from the first timepoint, respectively) were collected from JN16. Thirteen CHB patients were infected for at least 10 years (up to 25 years). The infection time could not be determined for the other three patients (JN09, JN11, and JN12). The average of viral loads was 4.51 × 10^7^ (9.41 × 10^2^ to 5.45 × 10^8^) IU/mL. HBsAg was positive for all 16 patients, while HBeAg was positive for 9 patients. Eight patients were treated with tenofovir disoproxil fumarate (TDF), but no TDF-associated resistance mutations were detected at the time when plasma samples were analyzed. In all four samples (JN13–JN16) which were followed for about 4 years, none were treated with any antiviral drugs. All patients were negative for HCV and HIV-1 infections. Only plasma samples were archived and available for this study from all 16 patients since they enrolled for studies in which only plasma was collected. All samples were stored at −80°C.

### Single genome amplification of near full-length HBV genomes

HBV genomic DNA was extracted from 200 µL plasma of each sample using the QIAamp DNA Mini Kit (Qiagen, Hilden, Germany). Single genome amplification (SGA) was performed to obtain NFL HBV genome sequences using Phanta Flash Master Mix (Vazyme, Nanjing, China) as previously reported (18). The first round of PCR was carried out with the primers P1 (5′-TTTTTCACCTCTGCCTAATCA-3′; nt 1,823–1,843) and P2 (5′-AAAAAGTTGCATGGTGCTGG-3′; nt 1,827–1,808), and the second round PCR was done with the primers NPP1 (5′-ACCTCTGCCTAATCATCTCTTGT-3′; nt 1,829–1,851) and NPP2 (5′-GTTGCATGGTGCTGGTGCGCAG-3′; nt 1,822–1,801. The PCR conditions for both rounds were as follows: an initial denaturation at 98°C for 30 s, followed by 35 cycles of denaturation at 98°C for 10 s, annealing at 58°C for 5 s, and extension at 72°C for 15 s, and a final extension at 72°C for 1 min.

### DNA sequencing

The PCR products (~3.2 kbp) were purified with the E.Z.N.A. Cycle-Pure Kit (Omega Bio-Tek, Georgia, USA) and subjected to cycle sequencing using BigDye terminator v3.1 (Applied Biosystems, Foster City, USA) on the ABI 3730XL DNA analyzer. Sequence contigs from each SGA were assembled and edited using Sequencher 5.4 (Gene Codes, Ann Arbor, USA). The final sequences were deposited in GenBank (accession numbers: PQ335234–PQ336013).

### Sequence analysis

The final assembled SGA sequences from each patient were aligned using the MAFFT version 7 (https://mafft.cbrc.jp/alignment/server/index.html). The resulting alignment was inspected using Seaview (62) and corrected around large indels when necessary. The consensus sequence was generated by aligning multiple viral sequences from the same patient, calculating the frequencies of the nucleotide or amino acid at each site in the alignment at the first timepoint, and selecting the base or amino acid with a frequency of ≥60% at each site. Highlighter mutation plots were generated using the Highlighter tool (18) (https://www.hiv.lanl.gov/content/sequence/HIGHLIGHT/highlighter_top.html). Sequences exhibiting significant hypersubstitutions (*P* < 0.1) and those enriched for APOBEC-mediated G-to-A substitutions were excluded from subsequent analysis utilizing the Hypermut tool (63) (https://www.hiv.lanl.gov/content/sequence/HYPERMUT/hypermut.html). The Maximum Likelihood phylogenetic tree was constructed using the GTR model via IQ-TREE (64) (https://www.hiv.lanl.gov/content/sequence/IQTREE/iqtree.html) or PhyML (65) (https://www.hiv.lanl.gov/content/sequence/PHYML/interface.html). The topology of the tree was assessed through 1,000 bootstrap replicates or Approximate Likelihood Ratio Test. Within-lineage genetic pairwise *P*-distances were computed using the sequences from each sample with MEGA v11 (66). HIV Sequence Database software (https://www.hiv.lanl.gov/content/sequence/ENTROPY/entropy.html) examines the Shannon entropy of viral quasispecies at different timepoints and uses randomization calculations to calculate statistical confidence. The accumulation of synonymous and nonsynonymous mutations for each of the four open reading frames (*PreC_Core*, *Pol*, *PreS1_S2_S*, and *X*) was assessed using the Synonymous Nonsynonymous Analysis Program (SNAP) tool (67) (https://www.hiv.lanl.gov/content/sequence/SNAP/SNAP.html) through pairwise comparison of all sequences within the four overlapping open reading frames of samples from different timepoints. Sites under positive selection, identified with a cutoff of *P* = 0.05, were determined utilizing the online Mixed Effects Model of Evolution tool from the Datamonkey server (68).

### Identification of potential immune escape mutations

Sequence fragments containing the MHR of HBsAg including the “a” determinant in the *S* gene from each patient contained the major target for nAbs and aligned. Any predominant and fixed mutation between different timepoints was examined to identify potential nAb escape mutations. NLG sites were analyzed with the tool from Los Alamos National laboratory websites (https://www.hiv.lanl.gov/content/sequence/GLYCOSITE/glycosite.html) (69) to identify changes in NLG sites. To identify potential T cell escape mutations, predominant and fixed mutations after HSS were examined against well-characterized T cell epitopes in the Hepitopes database (https://www.expmedndm.ox.ac.uk/hepitopes) (70), Immune Epitope Database (https://www.iedb.org) (71), and reported in previous studies (53, 72, 73).

### Evolutionary rate assessment

The sampling time of the sequences was used as a calibration point for the Bayesian Markov Chain Monte Carlo analysis to estimate the evolutionary rate of HBV in each patient. Constant coalescent, exponential coalescent, and Bayesian skyline coalescent tree priors were used with either a strict or a relaxed lognormal clock model. All models were run using the GTR + G4 substitution model in BEAST v2 (74) with a total Markov chain length set to 200 M, including a 10% burn-in. Path sampling was performed to select the best combination model. Convergence was assessed by ensuring that the effective sample size for each parameter was greater than 200.

### Statistical analysis

Statistical analysis was performed using SPSS 25.0. The Kruskal-Wallis test was used for multiple group analyses, and the Mann-Whitney test was used for comparisons between two groups. All tests were performed as two-sided tests, with *P* < 0.05 considered significant.

## Data Availability

The newly obtained sequences have been deposited in the GenBank under accession numbers PQ335234–PQ336013. All other data are presented in the manuscript.

## SUPPLEMENTAL MATERIAL

Fig. S1 mbio.01113-25-s0001.tif Maximum-likelihood phylogenetic analysis and genetic analysis of the near full-length HBV genome sequences from the CHB patients.

Fig. S2 mbio.01113-25-s0002.tif Genetic analysis of near full-length HBV genome sequences from the CHB patients.

Fig. S3 mbio.01113-25-s0003.tif Genetic evolution between two different timepoints in the CHB patients.

Fig. S4 mbio.01113-25-s0004.tif The role of recombination in the hard selective sweep.

Fig. S5 mbio.01113-25-s0005.tif Severe reduction of both synonymous and nonsynonymous mutations during the hard selective sweep.

Fig. S6 mbio.01113-25-s0006.tif Analysis of the accumulation of near full-length genome mutations in viruses from patients without HSS.

Fig. S7 mbio.01113-25-s0007.tif The predominant and fixed mutations are selected by the hard selective sweep.

Fig. S8 mbio.01113-25-s0008.tif Shannon entropy analysis of nearly full-length HBV sequences from 10 CHB patients at different timepoints.

Supplemental material mbio.01113-25-s0009.docx Tables S1–S3 and legends for Fig. S1–S8.

ASM does not own the copyrights to Supplemental Material that may be linked to, or accessed through, an article. The authors have granted ASM a non-exclusive, world-wide license to publish the Supplemental Material files. Please contact the corresponding author directly for reuse.

## References

[1] Terrault NA, Lok ASF, McMahon BJ, Chang K-M, Hwang JP, Jonas MM, Brown RS, Bzowej NH, Wong JB. 2018. Update on prevention, diagnosis, and treatment of chronic hepatitis B. Hepatology 67:1560–1599. doi:10.1002/hep.29800 29405329 PMC5975958

[2] Seeger C, Mason WS. 2000. Hepatitis B virus biology. Microbiol Mol Biol Rev 64:51–68. doi:10.1128/MMBR.64.1.51-68.2000 10704474 PMC98986

[3] Ganem D, Prince AM. 2004. Hepatitis B virus infection--natural history and clinical consequences. N Engl J Med 350:1118–1129. doi:10.1056/NEJMra031087 15014185

[4] Hepatitis B. Available from: https://www.who.int/news-room/fact-sheets/detail/hepatitis-b. Retrieved 16 Aug 2024.

[5] Domingo E, Gomez J. 2007. Quasispecies and its impact on viral hepatitis. Virus Res 127:131–150. doi:10.1016/j.virusres.2007.02.001 17349710 PMC7125676

[6] Betz-Stablein BD, Töpfer A, Littlejohn M, Yuen L, Colledge D, Sozzi V, Angus P, Thompson A, Revill P, Beerenwinkel N, Warner N, Luciani F. 2016. Single-molecule sequencing reveals complex genome variation of hepatitis B virus during 15 years of chronic infection following liver transplantation. J Virol 90:7171–7183. doi:10.1128/JVI.00243-16 27252524 PMC4984637

[7] Vignuzzi M, Stone JK, Arnold JJ, Cameron CE, Andino R. 2006. Quasispecies diversity determines pathogenesis through cooperative interactions in a viral population. Nature 439:344–348. doi:10.1038/nature04388 16327776 PMC1569948

[8] Farci P, Shimoda A, Coiana A, Diaz G, Peddis G, Melpolder JC, Strazzera A, Chien DY, Munoz SJ, Balestrieri A, Purcell RH, Alter HJ. 2000. The outcome of acute hepatitis C predicted by the evolution of the viral quasispecies. Science 288:339–344. doi:10.1126/science.288.5464.339 10764648

[9] Guo X, Jin Y, Qian G, Tu H. 2008. Sequential accumulation of the mutations in core promoter of hepatitis B virus is associated with the development of hepatocellular carcinoma in Qidong, China. J Hepatol 49:718–725. doi:10.1016/j.jhep.2008.06.026 18801591

[10] Raimondo G, Costantino L, Caccamo G, Pollicino T, Squadrito G, Cacciola I, Brancatelli S. 2004. Non-sequencing molecular approaches to identify preS2-defective hepatitis B virus variants proved to be associated with severe liver diseases. J Hepatol 40:515–519. doi:10.1016/j.jhep.2003.11.025 15123368

[11] Yeung P, Wong DK-H, Lai C-L, Fung J, Seto W-K, Yuen M-F. 2011. Association of hepatitis B virus pre-S deletions with the development of hepatocellular carcinoma in chronic hepatitis B. J Infect Dis 203:646–654. doi:10.1093/infdis/jiq096 21227916 PMC3072715

[12] Nie H, Evans AA, London WT, Block TM, Ren XD. 2012. Quantitative dynamics of hepatitis B basal core promoter and precore mutants before and after HBeAg seroconversion. J Hepatol 56:795–802. doi:10.1016/j.jhep.2011.11.012 22173170 PMC3307917

[13] Chen R, Liu Y, Luo D, Si L, Huang B, Wang J, Li X, Cheng F, Xu D, Duan C. 2020. Hepatitis B virus mutation pattern rtA181S+T184I+M204I may contribute to multidrug resistance in clinical practice: analysis of a large cohort of Chinese patients. Antiviral Res 180:104852. doi:10.1016/j.antiviral.2020.104852 32569703

[14] Oon CJ, Chen WN. 1998. Current aspects of hepatitis B surface antigen mutants in Singapore. J Viral Hepat 5 Suppl 2:17–23. doi:10.1046/j.1365-2893.1998.0050s2017.x 9857356

[15] Huang CH, Yuan Q, Chen PJ, Zhang YL, Chen CR, Zheng QB, Yeh SH, Yu H, Xue Y, Chen YX, Liu PG, Ge SX, Zhang J, Xia NS. 2012. Influence of mutations in hepatitis B virus surface protein on viral antigenicity and phenotype in occult HBV strains from blood donors. J Hepatol 57:720–729. doi:10.1016/j.jhep.2012.05.009 22634131

[16] Konopleva MV, Borisova VN, Sokolova MV, Semenenko TA, Suslov AP. 2022. Recombinant HBsAg of the wild-type and the G145R escape mutant, included in the new multivalent vaccine against hepatitis B virus, dramatically differ in their effects on leukocytes from healthy donors in vitro. Vaccines (Basel) 10:235. doi:10.3390/vaccines10020235 35214692 PMC8880183

[17] Palmer S, Kearney M, Maldarelli F, Halvas EK, Bixby CJ, Bazmi H, Rock D, Falloon J, Davey RT Jr, Dewar RL, Metcalf JA, Hammer S, Mellors JW, Coffin JM. 2005. Multiple, linked human immunodeficiency virus type 1 drug resistance mutations in treatment-experienced patients are missed by standard genotype analysis. J Clin Microbiol 43:406–413. doi:10.1128/JCM.43.1.406-413.2005 15635002 PMC540111

[18] Keele BF, Giorgi EE, Salazar-Gonzalez JF, Decker JM, Pham KT, Salazar MG, Sun C, Grayson T, Wang S, Li H, et al.. 2008. Identification and characterization of transmitted and early founder virus envelopes in primary HIV-1 infection. Proc Natl Acad Sci USA 105:7552–7557. doi:10.1073/pnas.0802203105 18490657 PMC2387184

[19] Liu SL, Rodrigo AG, Shankarappa R, Learn GH, Hsu L, Davidov O, Zhao LP, Mullins JI. 1996. HIV quasispecies and resampling. Science 273:415–416. doi:10.1126/science.273.5274.415 8677432

[20] Salk JJ, Schmitt MW, Loeb LA. 2018. Enhancing the accuracy of next-generation sequencing for detecting rare and subclonal mutations. Nat Rev Genet 19:269–285. 29576615 10.1038/nrg.2017.117 PMC6485430

[21] Salazar-Gonzalez JF, Bailes E, Pham KT, Salazar MG, Guffey MB, Keele BF, Derdeyn CA, Farmer P, Hunter E, Allen S, Manigart O, Mulenga J, Anderson JA, Swanstrom R, Haynes BF, Athreya GS, Korber BTM, Sharp PM, Shaw GM, Hahn BH. 2008. Deciphering human immunodeficiency virus type 1 transmission and early envelope diversification by single-genome amplification and sequencing. J Virol 82:3952–3970. 18256145 10.1128/JVI.02660-07 PMC2293010

[22] Meyerhans A, Vartanian JP, Wain-Hobson S. 1990. DNA recombination during PCR. Nucleic Acids Res 18:1687–1691. doi:10.1093/nar/18.7.1687 2186361 PMC330584

[23] Liu J, Song H, Liu D, Zuo T, Lu F, Zhuang H, Gao F. 2014. Extensive recombination due to heteroduplexes generates large amounts of artificial gene fragments during PCR. PLoS ONE 9:e106658. doi:10.1371/journal.pone.0106658 25211143 PMC4161356

[24] Thai H, Campo DS, Lara J, Dimitrova Z, Ramachandran S, Xia G, Ganova-Raeva L, Teo C-G, Lok A, Khudyakov Y. 2012. Convergence and coevolution of hepatitis B virus drug resistance. Nat Commun 3:789. doi:10.1038/ncomms1794 22510694 PMC3337990

[25] Liao H-X, Lynch R, Zhou T, Gao F, Alam SM, Boyd SD, Fire AZ, Roskin KM, Schramm CA, Zhang Z, et al.. 2013. Co-evolution of a broadly neutralizing HIV-1 antibody and founder virus. Nature 496:469–476. doi:10.1038/nature12053 23552890 PMC3637846

[26] Ke R, Li H, Wang S, Ding W, Ribeiro RM, Giorgi EE, Bhattacharya T, Barnard RJO, Hahn BH, Shaw GM, Perelson AS. 2018. Superinfection and cure of infected cells as mechanisms for hepatitis C virus adaptation and persistence. Proc Natl Acad Sci USA 115:E7139–E7148. doi:10.1073/pnas.1805267115 29987026 PMC6065014

[27] Liu W, Li Y, Learn GH, Rudicell RS, Robertson JD, Keele BF, Ndjango J-B, Sanz CM, Morgan DB, Locatelli S, Gonder MK, Kranzusch PJ, Walsh PD, Delaporte E, Mpoudi-Ngole E, Georgiev AV, Muller MN, Shaw GM, Peeters M, Sharp PM, Rayner JC, Hahn BH. 2010. Origin of the human malaria parasite Plasmodium falciparum in gorillas. Nature 467:420–425. doi:10.1038/nature09442 20864995 PMC2997044

[28] Gao F, Bonsignori M, Liao H-X, Kumar A, Xia S-M, Lu X, Cai F, Hwang K-K, Song H, Zhou T, et al.. 2014. Cooperation of B cell lineages in induction of HIV-1-broadly neutralizing antibodies. Cell 158:481–491. doi:10.1016/j.cell.2014.06.022 25065977 PMC4150607

[29] Smith JM, Haigh J. 1974. The hitch-hiking effect of a favourable gene. Genet Res 23:23–35. 4407212

[30] Stephan W. 2019. Selective sweeps. Genetics 211:5–13. doi:10.1534/genetics.118.301319 30626638 PMC6325696

[31] Hermisson J, Pennings PS. 2005. Soft sweeps. Genetics 169:2335–2352. doi:10.1534/genetics.104.036947 15716498 PMC1449620

[32] Goonetilleke N, Liu MKP, Salazar-Gonzalez JF, Ferrari G, Giorgi E, Ganusov VV, Keele BF, Learn GH, Turnbull EL, Salazar MG, Weinhold KJ, Moore S, Letvin N, Haynes BF, Cohen MS, Hraber P, Bhattacharya T, Borrow P, Perelson AS, Hahn BH, Shaw GM, Korber BT, McMichael AJ, CHAVI Clinical Core B. 2009. The first T cell response to transmitted/founder virus contributes to the control of acute viremia in HIV-1 infection. J Exp Med 206:1253–1272. doi:10.1084/jem.20090365 19487423 PMC2715063

[33] Bull RA, Luciani F, McElroy K, Gaudieri S, Pham ST, Chopra A, Cameron B, Maher L, Dore GJ, White PA, Lloyd AR. 2011. Sequential bottlenecks drive viral evolution in early acute hepatitis C virus infection. PLoS Pathog 7:e1002243. doi:10.1371/journal.ppat.1002243 21912520 PMC3164670

[34] Kang L, He G, Sharp AK, Wang X, Brown AM, Michalak P, Weger-Lucarelli J. 2021. A selective sweep in the Spike gene has driven SARS-CoV-2 human adaptation. Cell 184:4392–4400. doi:10.1016/j.cell.2021.07.007 34289344 PMC8260498

[35] Rambaut A, Pybus OG, Nelson MI, Viboud C, Taubenberger JK, Holmes EC. 2008. The genomic and epidemiological dynamics of human influenza A virus. Nature 453:615–619. doi:10.1038/nature06945 18418375 PMC2441973

[36] Li H, Stoddard MB, Wang S, Blair LM, Giorgi EE, Parrish EH, Learn GH, Hraber P, Goepfert PA, Saag MS, Denny TN, Haynes BF, Hahn BH, Ribeiro RM, Perelson AS, Korber BT, Bhattacharya T, Shaw GM. 2012. Elucidation of hepatitis C virus transmission and early diversification by single genome sequencing. PLoS Pathog 8:e1002880. doi:10.1371/journal.ppat.1002880 22927816 PMC3426529

[37] Feder AF, Rhee S-Y, Holmes SP, Shafer RW, Petrov DA, Pennings PS. 2016. More effective drugs lead to harder selective sweeps in the evolution of drug resistance in HIV-1. Elife 5:e10670. doi:10.7554/eLife.10670 26882502 PMC4764592

[38] Pennings PS, Kryazhimskiy S, Wakeley J. 2014. Loss and recovery of genetic diversity in adapting populations of HIV. PLoS Genet 10:e1004000. doi:10.1371/journal.pgen.1004000 24465214 PMC3900388

[39] van Zyl G, Bale MJ, Kearney MF. 2018. HIV evolution and diversity in ART-treated patients. Retrovirology (Auckl) 15:14. doi:10.1186/s12977-018-0395-4 PMC5789667 29378595

[40] Yeo YH, Ho HJ, Yang H-I, Tseng T-C, Hosaka T, Trinh HN, Kwak M-S, Park YM, Fung JYY, Buti M, et al.. 2019. Factors associated with rates of HBsAg seroclearance in adults with chronic HBV infection: a systematic review and meta-analysis. Gastroenterology 156:635–646. doi:10.1053/j.gastro.2018.10.027 30342034

[41] Kim KH, Chang HY, Park JY, Park ES, Park YK, Han KH, Ahn SH. 2014. Spontaneous HBsAg loss in Korean patients: relevance of viral genotypes, S gene mutations, and covalently closed circular DNA copy numbers. Clin Mol Hepatol 20:251. doi:10.3350/cmh.2014.20.3.251 25320728 PMC4197173

[42] Liu J, Yang H-I, Lee M-H, Lu S-N, Jen C-L, Wang L-Y, You S-L, Iloeje UH, Chen C-J. 2010. Incidence and determinants of spontaneous hepatitis B surface antigen seroclearance: a community-based follow-up study. Gastroenterology 139:474–482. doi:10.1053/j.gastro.2010.04.048 20434450

[43] Wang H-Y, Chien M-H, Huang H-P, Chang H-C, Wu C-C, Chen P-J, Chang M-H, Chen D-S. 2010. Distinct hepatitis B virus dynamics in the immunotolerant and early immunoclearance phases. J Virol 84:3454–3463. doi:10.1128/JVI.02164-09 20089644 PMC2838120

[44] Shen T, Yan X-M, Zhang J-P, Wang J-L, Zuo R-X, Li L, Wang L-P. 2011. Evolution of hepatitis B virus in a chronic HBV-infected patient over 2 years. Hepat Res Treat 2011:1–6. doi:10.1155/2011/939148 PMC3139125 21785721

[45] Ye B, Liu X, Li X, Kong H, Tian L, Chen Y. 2015. T-cell exhaustion in chronic hepatitis B infection: current knowledge and clinical significance. Cell Death Dis 6:e1694–e1694. doi:10.1038/cddis.2015.42 25789969 PMC4385920

[46] Burton AR, Pallett LJ, McCoy LE, Suveizdyte K, Amin OE, Swadling L, Alberts E, Davidson BR, Kennedy PTF, Gill US, Mauri C, Blair PA, Pelletier N, Maini MK. 2018. Circulating and intrahepatic antiviral B cells are defective in hepatitis B. J Clin Invest 128:4588–4603. doi:10.1172/JCI121960 30091725 PMC6159997

[47] Dusheiko GM, Hoofnagle JH, Cooksley WG, James SP, Jones EA. 1983. Synthesis of antibodies to hepatitis B virus by cultured lymphocytes from chronic hepatitis B surface antigen carriers. J Clin Invest 71:1104–1113. doi:10.1172/jci110860 6602149 PMC436971

[48] McLane LM, Abdel-Hakeem MS, Wherry EJ. 2019. CD8 T cell exhaustion during chronic viral infection and cancer. Annu Rev Immunol 37:457–495. doi:10.1146/annurev-immunol-041015-055318 30676822

[49] Barnaba V, Franco A, Alberti A, Benvenuto R, Balsano F. 1990. Selective killing of hepatitis B envelope antigen-specific B cells by class I-restricted, exogenous antigen-specific T lymphocytes. Nature 345:258–260. doi:10.1038/345258a0 2110296

[50] Lazarevic I, Banko A, Miljanovic D, Cupic M. 2019. Immune-escape hepatitis B virus mutations associated with viral reactivation upon immunosuppression. Viruses 11:778. doi:10.3390/v11090778 31450544 PMC6784188

[51] Boni C, Fisicaro P, Valdatta C, Amadei B, Di Vincenzo P, Giuberti T, Laccabue D, Zerbini A, Cavalli A, Missale G, Bertoletti A, Ferrari C. 2007. Characterization of hepatitis B virus (HBV)-specific T-cell dysfunction in chronic HBV infection. J Virol 81:4215–4225. doi:10.1128/JVI.02844-06 17287266 PMC1866111

[52] Bertoletti A, Ferrari C. 2016. Adaptive immunity in HBV infection. J Hepatol 64:S71–S83. doi:10.1016/j.jhep.2016.01.026 27084039

[53] Wu Y, Ding Y, Shen C. 2022. A systematic review of T cell epitopes defined from the proteome of hepatitis B virus. Vaccines (Basel) 10:257. doi:10.3390/vaccines10020257 35214714 PMC8878595

[54] Mühlemann B, Jones TC, Damgaard P de B, Allentoft ME, Shevnina I, Logvin A, Usmanova E, Panyushkina IP, Boldgiv B, Bazartseren T, et al.. 2018. Ancient hepatitis B viruses from the Bronze Age to the Medieval period. Nature 557:418–423. doi:10.1038/s41586-018-0097-z 29743673

[55] Orito E, Mizokami M, Ina Y, Moriyama EN, Kameshima N, Yamamoto M, Gojobori T. 1989. Host-independent evolution and a genetic classification of the hepadnavirus family based on nucleotide sequences. Proc Natl Acad Sci USA 86:7059–7062. doi:10.1073/pnas.86.18.7059 2780562 PMC297993

[56] Girones R, Miller RH. 1989. Mutation rate of the hepadnavirus genome. Virology (Auckl) 170:595–597. doi:10.1016/0042-6822(89)90455-8 2728351

[57] Fares MA, Holmes EC. 2002. A revised evolutionary history of hepatitis B virus (HBV). J Mol Evol 54:807–814. doi:10.1007/s00239-001-0084-z 12029362

[58] Osiowy C, Giles E, Tanaka Y, Mizokami M, Minuk GY. 2006. Molecular evolution of hepatitis B virus over 25 years. J Virol 80:10307–10314. doi:10.1128/JVI.00996-06 17041211 PMC1641782

[59] Zhou Y, Holmes EC. 2007. Bayesian estimates of the evolutionary rate and age of hepatitis B virus. J Mol Evol 65:197–205. doi:10.1007/s00239-007-0054-1 17684696

[60] Simonetti J, Bulkow L, McMahon BJ, Homan C, Snowball M, Negus S, Williams J, Livingston SE. 2010. Clearance of hepatitis B surface antigen and risk of hepatocellular carcinoma in a cohort chronically infected with hepatitis B virus. Hepatology 51:1531–1537. doi:10.1002/hep.23464 20087968

[61] Song H, Giorgi EE, Ganusov VV, Cai F, Athreya G, Yoon H, Carja O, Hora B, Hraber P, Romero-Severson E, et al.. 2018. Tracking HIV-1 recombination to resolve its contribution to HIV-1 evolution in natural infection. Nat Commun 9:1928. doi:10.1038/s41467-018-04217-5 29765018 PMC5954121

[62] Gouy M, Guindon S, Gascuel O. 2010. SeaView version 4: a multiplatform graphical user interface for sequence alignment and phylogenetic tree building. Mol Biol Evol 27:221–224. doi:10.1093/molbev/msp259 19854763

[63] Rose PP, Korber BT. 2000. Detecting hypermutations in viral sequences with an emphasis on G --> A hypermutation. Bioinformatics 16:400–401. doi:10.1093/bioinformatics/16.4.400 10869039

[64] Trifinopoulos J, Nguyen L-T, von Haeseler A, Minh BQ. 2016. W-IQ-TREE: a fast online phylogenetic tool for maximum likelihood analysis. Nucleic Acids Res 44:W232–W235. doi:10.1093/nar/gkw256 27084950 PMC4987875

[65] Guindon S, Dufayard J-F, Lefort V, Anisimova M, Hordijk W, Gascuel O. 2010. New algorithms and methods to estimate maximum-likelihood phylogenies: assessing the performance of PhyML 3.0. Syst Biol 59:307–321. doi:10.1093/sysbio/syq010 20525638

[66] Tamura K, Stecher G, Kumar S. 2021. MEGA11: molecular evolutionary genetics analysis version 11. Mol Biol Evol 38:3022–3027. doi:10.1093/molbev/msab120 33892491 PMC8233496

[67] HIV signature and sequence variation analysis. Computational analysis of HIV molecular sequences. ScienceOpen. Available from: https://www.scienceopen.com/document?vid=f054582b-15a6-4ccc-be34-99d0e9d09004. Retrieved 22 Jul 2024.

[68] Murrell B, Wertheim JO, Moola S, Weighill T, Scheffler K, Kosakovsky Pond SL. 2012. Detecting individual sites subject to episodic diversifying selection. PLoS Genet 8:e1002764. doi:10.1371/journal.pgen.1002764 22807683 PMC3395634

[69] Zhang M, Gaschen B, Blay W, Foley B, Haigwood N, Kuiken C, Korber B. 2004. Tracking global patterns of N-linked glycosylation site variation in highly variable viral glycoproteins: HIV, SIV, and HCV envelopes and influenza hemagglutinin. Glycobiology 14:1229–1246. doi:10.1093/glycob/cwh106 15175256

[70] Lumley S, Noble H, Hadley MJ, Callow L, Malik A, Chua YY, Duffey OJ, Grolmusova N, Kumar A, Ravenscroft S, Spencer JI, Neumann-Haefelin C, Thimme R, Andersson M, Klenerman P, Barnes E, Matthews PC. 2016. Hepitopes: a live interactive database of HLA class I epitopes in hepatitis B virus. Wellcome Open Res 1:9. doi:10.12688/wellcomeopenres.9952.1 27976751 PMC5142601

[71] Schwarz T, Ptok J, Damagnez M, Menne C, Alizei ES, Lang-Meli J, Maas M, Habermann D, Hoffmann D, Schulze zur Wiesch J, et al.. 2025. HBV shows different levels of adaptation to HLA class I-associated selection pressure correlating with markers of replication. J Hepatol 82:805–815. doi:10.1016/j.jhep.2024.10.047 39536821

[72] de Beijer MTA, Jansen DTSL, Dou Y, van Esch WJE, Mok JY, Maas MJP, Brasser G, de Man RA, Woltman AM, Buschow SI. 2020. Discovery and selection of hepatitis B virus-derived T cell epitopes for global immunotherapy based on viral indispensability, conservation, and HLA-binding strength. J Virol 94:e01663-19. doi:10.1128/JVI.01663-19 31852786 PMC7081907

[73] Cheng Y, Zhu YO, Becht E, Aw P, Chen J, Poidinger M, de Sessions PF, Hibberd ML, Bertoletti A, Lim SG, Newell EW. 2019. Multifactorial heterogeneity of virus-specific T cells and association with the progression of human chronic hepatitis B infection. Sci Immunol 4:eaau6905. doi:10.1126/sciimmunol.aau6905 30737354

[74] Suchard MA, Lemey P, Baele G, Ayres DL, Drummond AJ, Rambaut A. 2018. Bayesian phylogenetic and phylodynamic data integration using BEAST 1.10. Virus Evol 4:vey016. doi:10.1093/ve/vey016 29942656 PMC6007674

