# Supplementary figures and images for: Near extinction of the HBV quasispecies driven by the hard selective sweep in chronic hepatitis B
Source: mBio. 2025 Jun 30;16(8):e01113-25. doi: 10.1128/mbio.01113-25 (PMC12345218; doi:10.1128/mbio.01113-25)

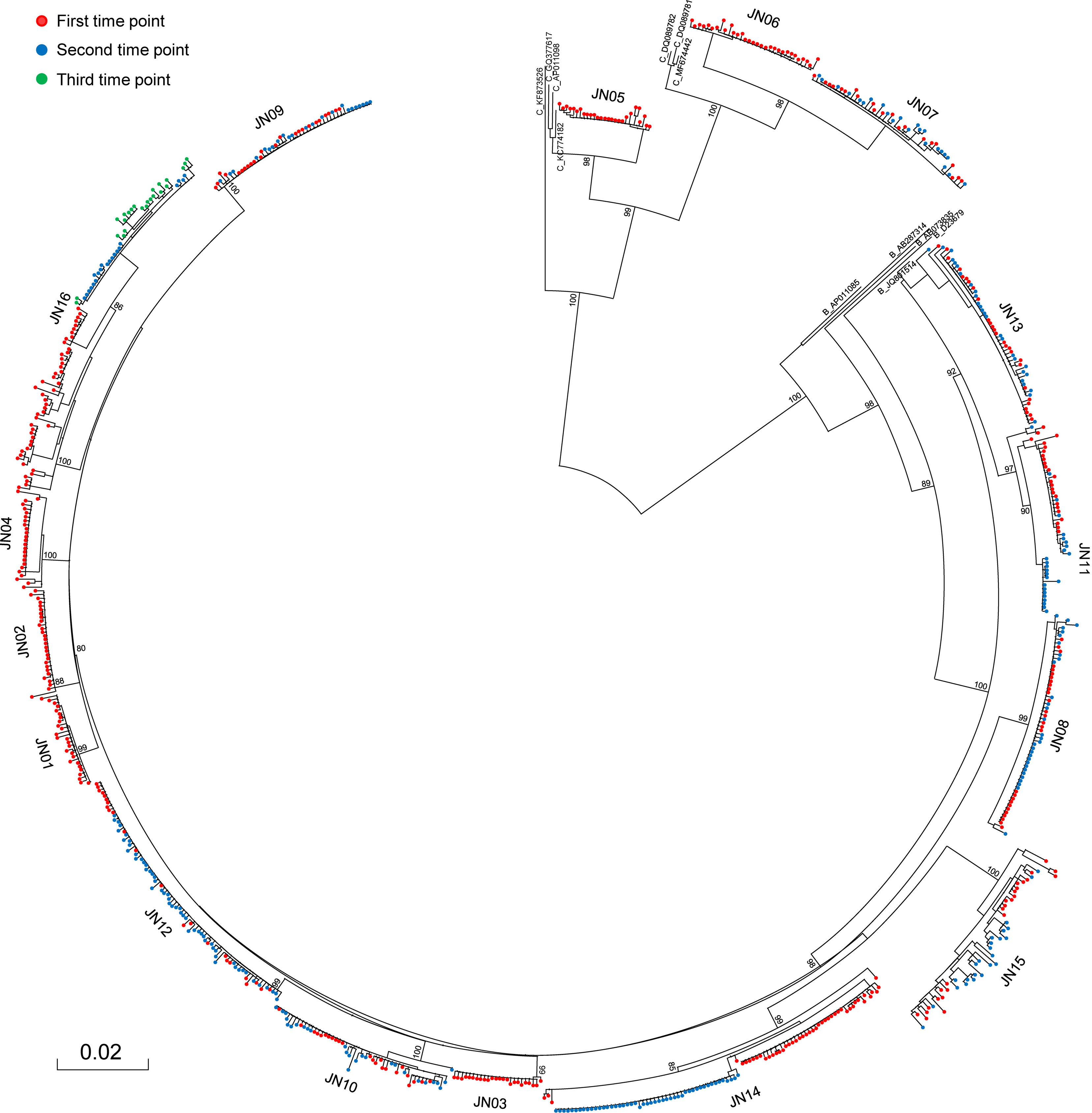

Supplement: Fig. S1 — Maximum-likelihood phylogenetic analysis and genetic analysis of the near full-length HBV genome sequences from the CHB patients. [file mbio.01113-25-s0001.tif]

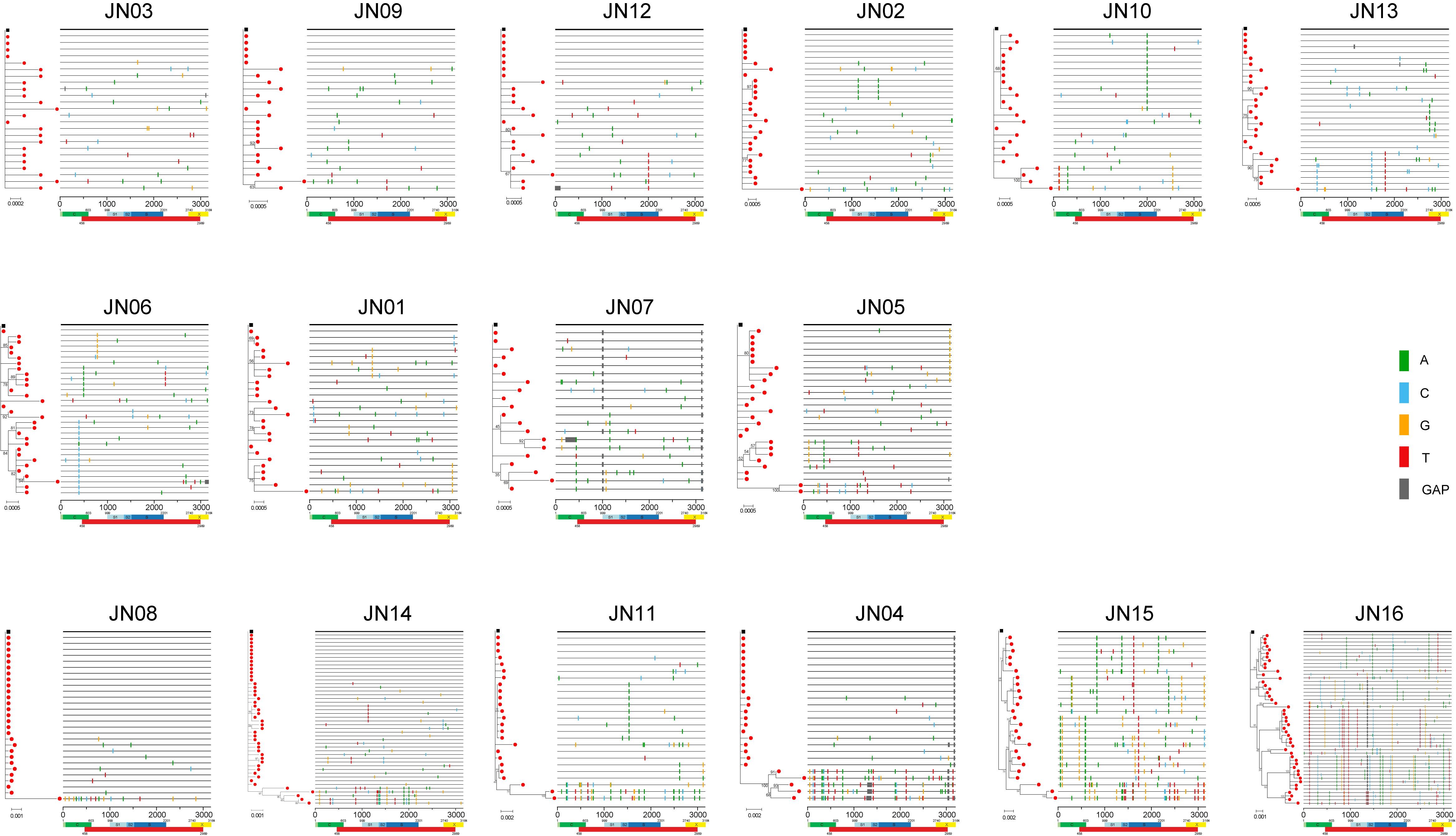

Supplement: Fig. S2 — Genetic analysis of near full-length HBV genome sequences from the CHB patients. [file mbio.01113-25-s0002.tif]

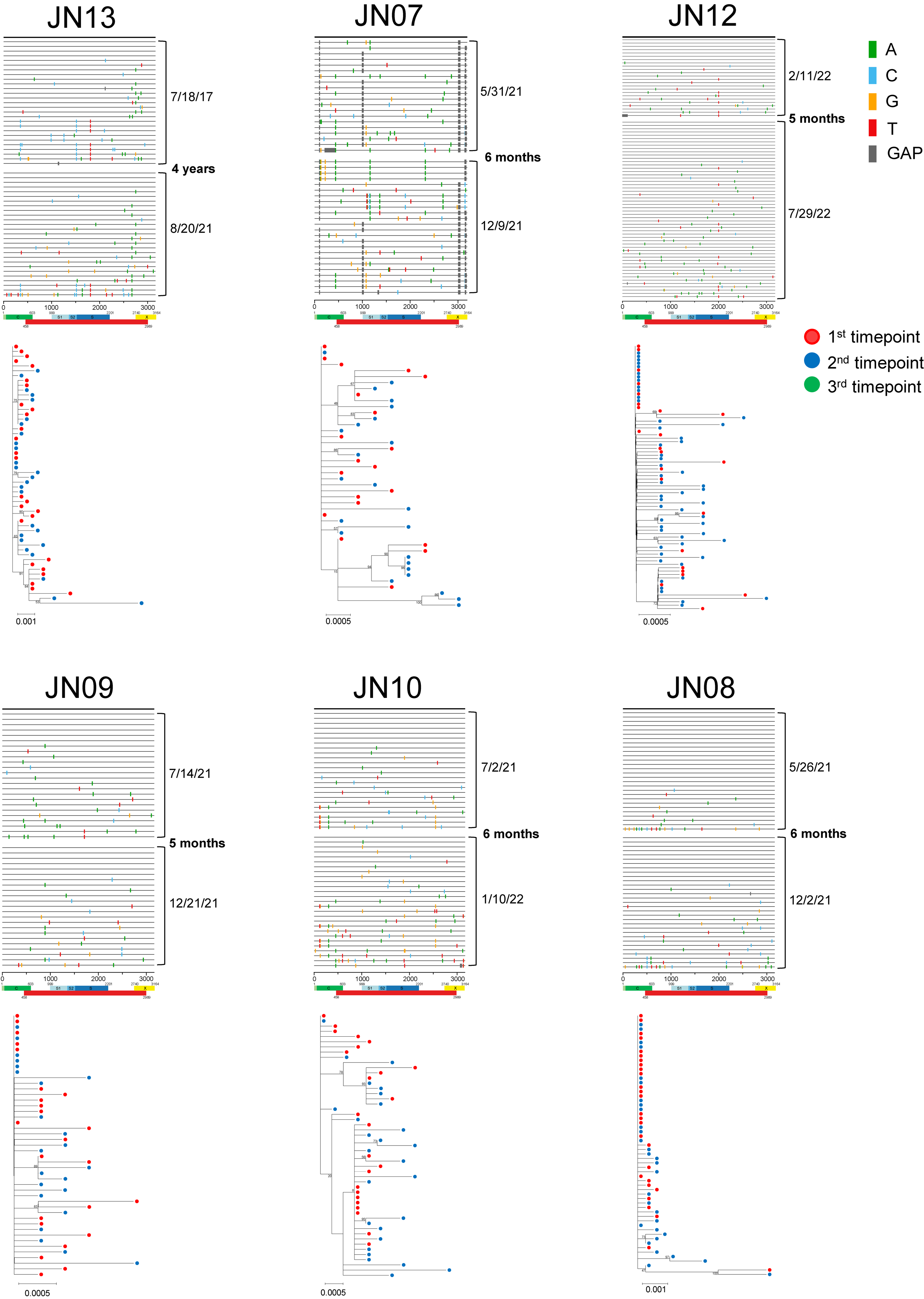

Supplement: Fig. S3 — Genetic evolution between two different timepoints in the CHB patients. [file mbio.01113-25-s0003.tif]

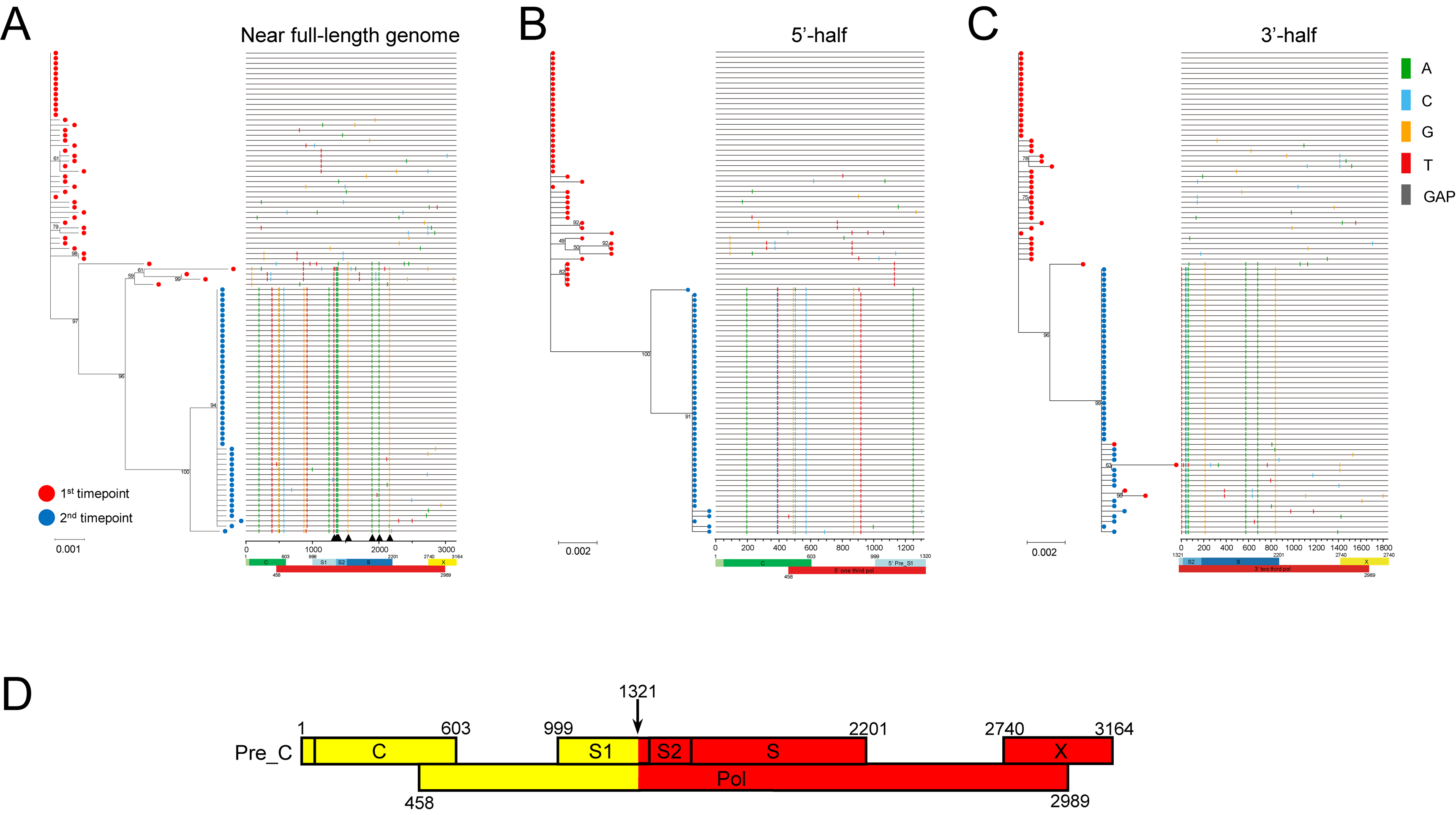

Supplement: Fig. S4 — The role of recombination in the hard selective sweep. [file mbio.01113-25-s0004.tif]

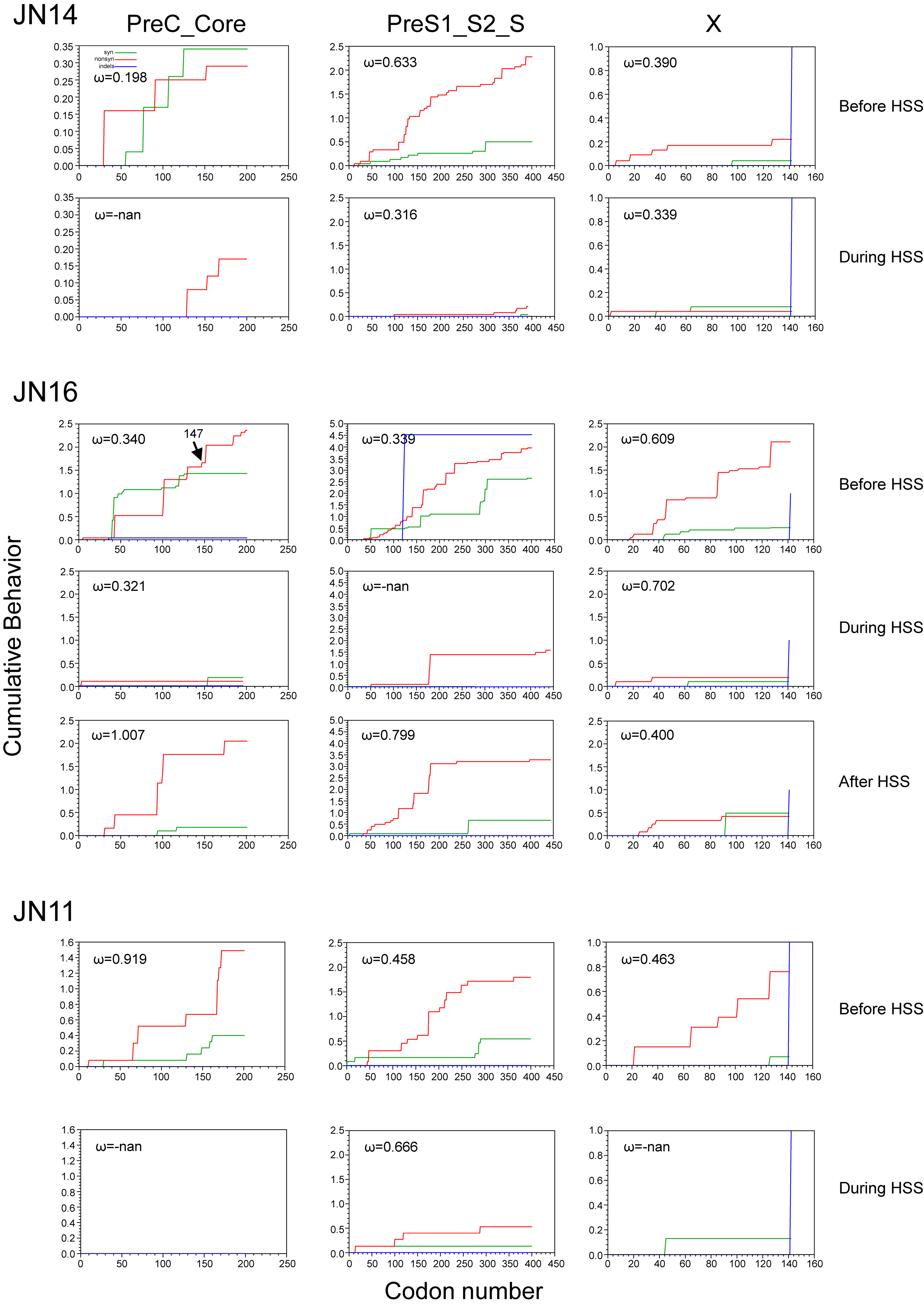

Supplement: Fig. S5 — Severe reduction of both synonymous and nonsynonymous mutations during the hard selective sweep. [file mbio.01113-25-s0005.tif]

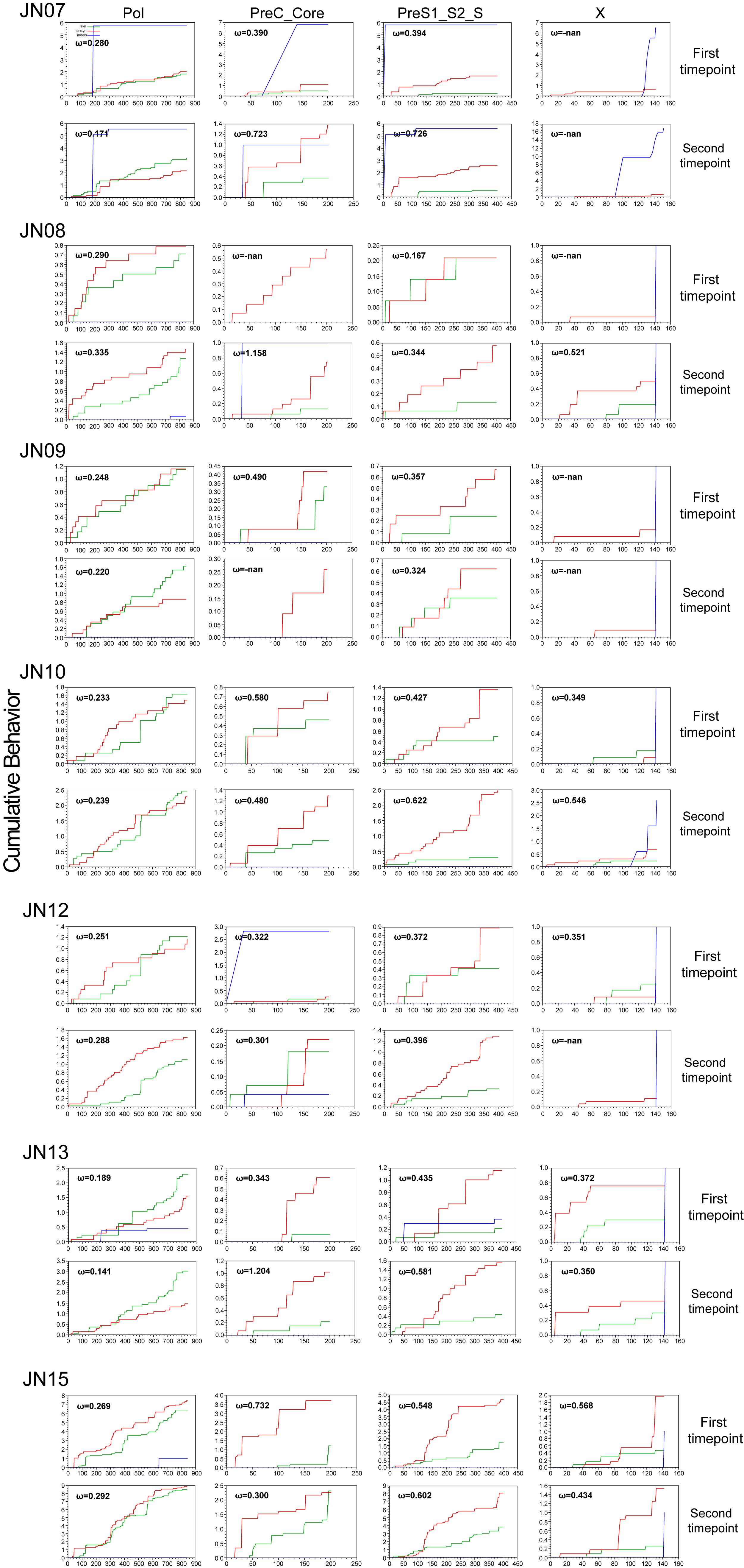

Supplement: Fig. S6 — Analysis of the accumulation of near full-length genome mutations in viruses from patients without HSS. [file mbio.01113-25-s0006.tif]

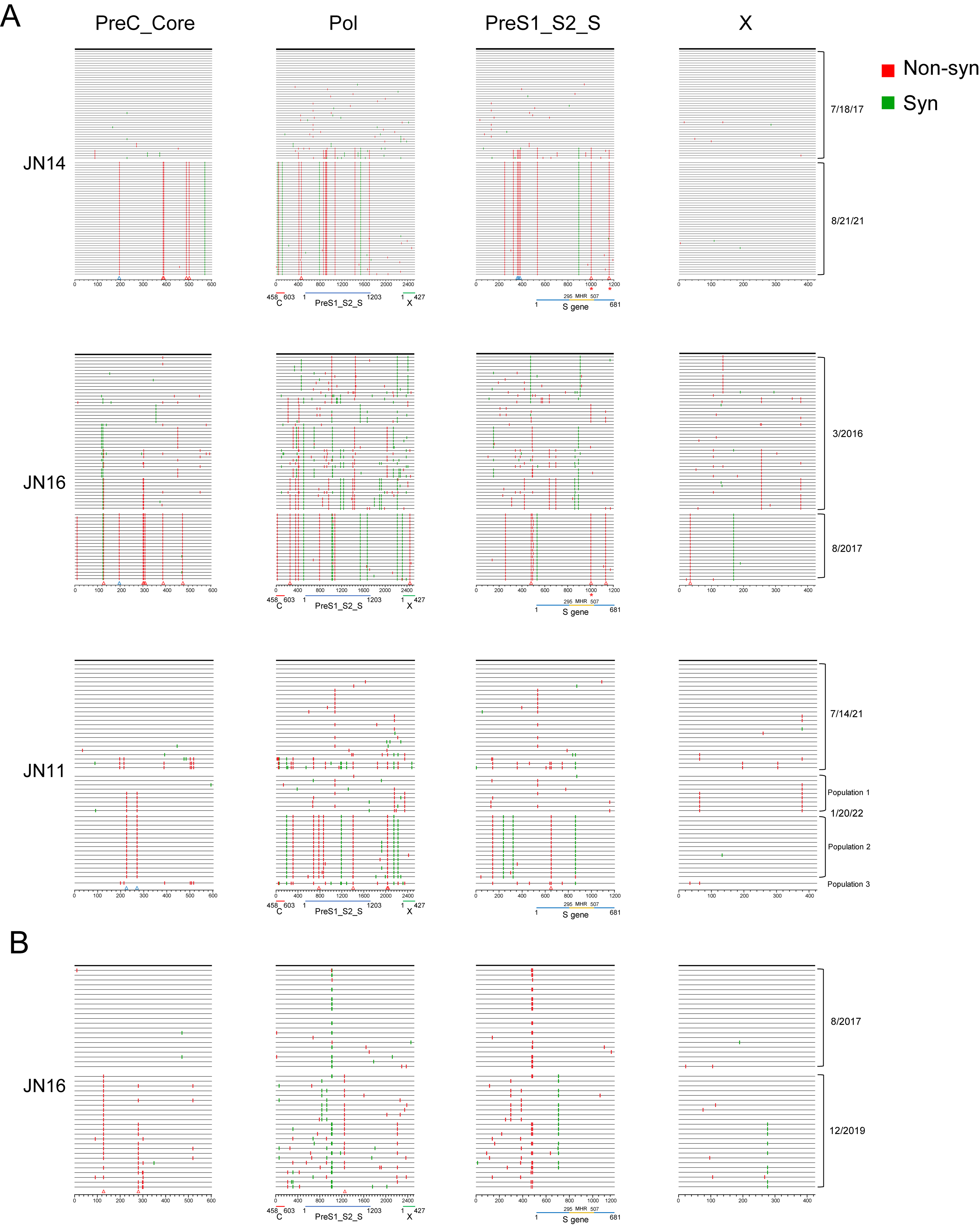

Supplement: Fig. S7 — The predominant and fixed mutations are selected by the hard selective sweep. [file mbio.01113-25-s0007.tif]

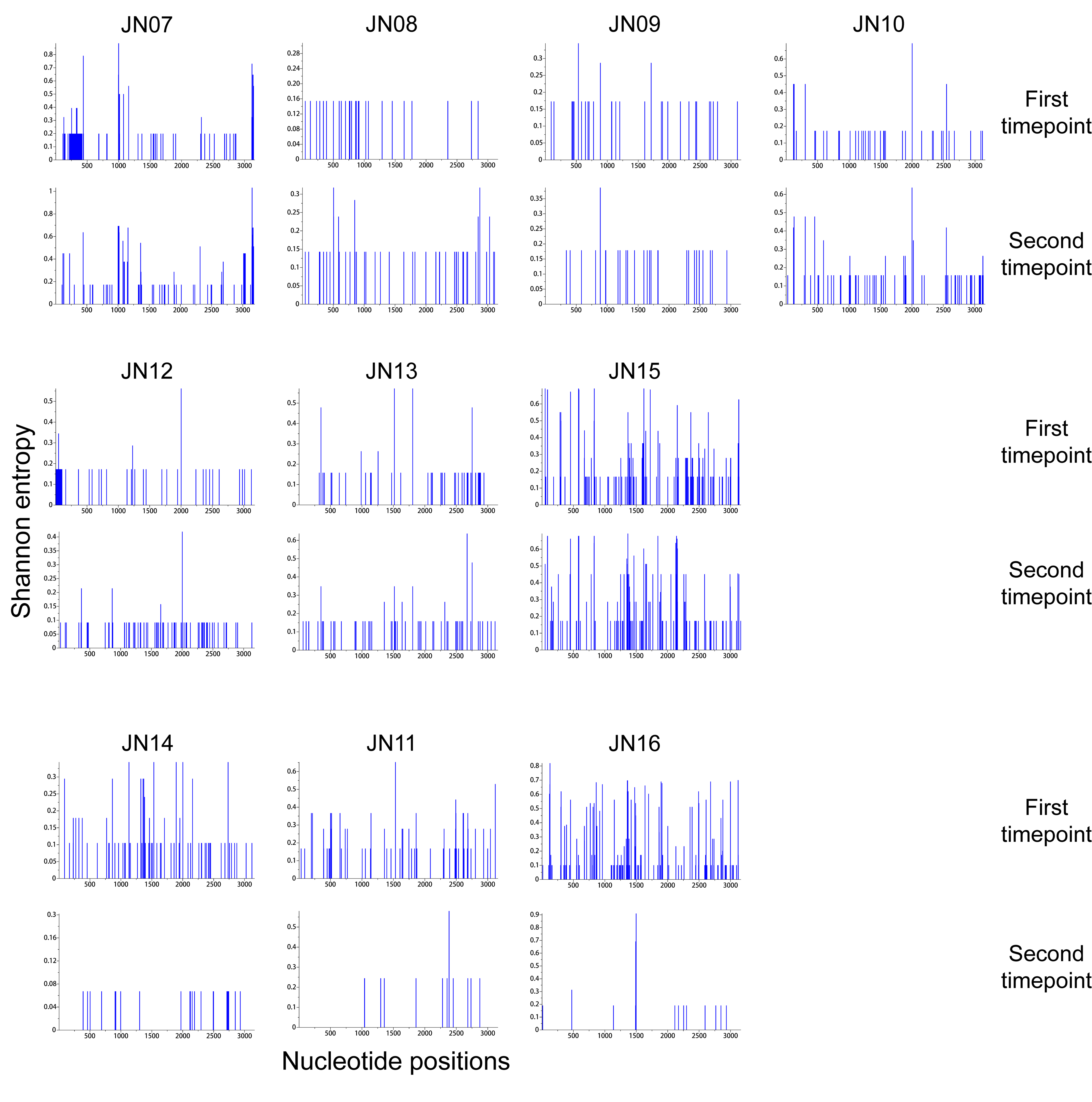

Supplement: Fig. S8 — Shannon entropy analysis of nearly full-length HBV sequences from 10 CHB patients at different timepoints. [file mbio.01113-25-s0008.tif]
