# Supplementary material for: Near extinction of the HBV quasispecies driven by the hard selective sweep in chronic hepatitis B
Source: mBio. 2025 Jun 30;16(8):e01113-25. doi: 10.1128/mbio.01113-25 (PMC12345218; doi:10.1128/mbio.01113-25)
Supplement: Supplemental material — Tables S1–S3 and legends for Fig. S1–S8. [file mbio.01113-25-s0009.docx]

**Supplementary materials**

**Near extinction of diverse HBV quasispecies driven by the hard selective sweep during chronic hepatitis B**

Daiqiang Lu^1, #^, Andong He^2, #^, Guichan Liao^3, #^, Renyu Zhou^1, #^, Zichun Cheng^1^, Ka Cheuk Yip^2^, Xiufang Wang^2^, Wei Cao^1^, Jiaojiao Peng^1^, Ruiman Li^2,^ *, Jie Peng^3,^ *, Feng Gao^1,4,^ *

^1^Institute of Molecular and Medical Virology, School of Medicine, Jinan University, Guangzhou, 510632, China.

^2^Department of Obstetrics and Gynecology, The First Affiliated Hospital of Jinan University, Guangzhou, 510630, China.

^3^Department of Infectious Diseases, Nanfang Hospital, Southern Medical University, Guangzhou, 510080, China.

^4^Key Laboratory of Viral Pathogenesis & Infection Prevention and Control, School of Medicine, Jinan University, Guangzhou, 510632, China.

^#^These authors contributed equally.

*Corresponding authors

fenggao@jnu.edu.cn (F. Gao); Pjie138@163.com (Jie. Peng); ruimanli@jnu.edu.cn (R. Li)

**Table S1. Demographics and characteristics of patients.**

| **PID** | **Gender** | **Age (years)** | **Sample collection date** | **Viral load (IU/mL)** | **HBsAg** | **HBeAg** | **Period of infection (year)** | **Antiviral treatment drug** | **genotype** | **No. of SGSs** |
| --- | --- | --- | --- | --- | --- | --- | --- | --- | --- | --- |
|  |  |  |  |  |  |  |  |  |  |  |
| JN01 | Female | 32 | 5/21/22 | 3.36×10^7^ | + | + | >10 | TDF | B | 26 |
| JN02 | Female | 35 | 6/10/22 | 4.07×10^7^ | + | + | 25 | TDF | B | 28 |
| JN03 | Female | 26 | 5/2/22 | 2.07×10^7^ | + | + | >10 | No | B | 24 |
| JN04 | Female | 29 | 5/2/22 | 6.62×10^3^ | + | + | 10 | TDF | B | 25 |
| JN05 | Female | 28 | 11/30/21 | 3.38×10^7^ | + | + | 11 | TDF | C | 27 |
| JN06 | Female | 36 | 12/9/21 | 3.42×10^6^ | + | - | 21 | TDF | C | 31 |
| JN07 | Female | 30 | 5/31/21 | 5.45×10^8^ | + | + | 10 | No | C | 20 |
|  |  |  | 12/9/21 | NA | NA | NA |  | TDF |  | 24 |
| JN08 | Female | 32 | 5/26/21 | 9.41×10^2^ | + | - | 14 | No | B | 28 |
|  |  |  | 12/2/21 | 1.90×10^3^ | + | - |  | No |  | 31 |
| JN09 | Female | 28 | 7/14/21 | NA | + | + | Unknown | No | B | 24 |
|  |  |  | 12/21/21 | 5.88×10^7^ | + | + |  | No |  | 23 |
| JN10 | Female | 28 | 7/2/21 | NA | + | + | >10 | No | B | 24 |
|  |  |  | 1/10/22 | 1.62×10^4^ | + | + |  | TDF |  | 27 |
| JN11 | Female | 27 | 7/14/21 | 2.61×10^4^ | + | + | Unknown | No | B | 25 |
|  |  |  | 1/20/22 | 3.25×10^4^ | + | + |  | No |  | 25 |
| JN12 | Female | 26 | 2/11/22 | 2.14×10^8^ | + | + | Unknown | No | B | 24 |
|  |  |  | 7/29/22 | 1.32×10^8^ | + | + |  | TDF |  | 54 |
| JN13 | Female | 36 | 7/18/17 | 5.14×10^4^ | + | - | >10 | No | B | 27 |
|  |  | 40 | 8/20/21 | 6.10×10^5^ | + | - |  | No |  | 27 |
| JN14 | Male | 24 | 7/18/17 | 2.11×10^3^ | + | - | >10 | No | B | 46 |
|  |  | 28 | 8/21/21 | 2.97×10^4^ | + | - |  | No |  | 48 |
| JN15 | Male | 41 | 8/2019 | 2.02×10^4^ | + | - | >10 | No | B | 25 |
|  |  | 46 | 4/2024 | 8.71×10^4^ | + | - |  | No |  | 24 |
| JN16 | Male | 42 | 3/2016 | 4.56×10^3^ | + | - | >10 | No | B | 48 |
|  |  | 43 | 8/2017 | 8.58×10^3^ | + | - |  | No |  | 21 |
|  |  | 45 | 12/2019 | 1.61×10^3^ | + | - |  | No |  | 24 |

TDF, tenofovir disoproxil fumarate; SGS, single genome sequencing; NA, not available.

When the participants reported a childhood diagnosis of HBV infection, their infection time was considered as more than 10 years.

**Table S2. intra-host genetic diversity of the viral populations at different timepoints in the CHB patients.**

|  | 1^st^ | 2^nd^ | 3^rd^ | Time interval (yr) |
| --- | --- | --- | --- | --- |
| JN01 | 0.16 | NA | NA | NA |
| JN02 | 0.12 | NA | NA | NA |
| JN03 | 0.08 | NA | NA | NA |
| JN04 | 0.27 | NA | NA | NA |
| JN05 | 0.19 | NA | NA | NA |
| JN06 | 0.15 | NA | NA | NA |
| JN07 | 0.18 | 0.24 | NA | 0.5 |
| JN08 | 0.06 | 0.11 | NA | 0.5 |
| JN09 | 0.09 | 0.08 | NA | 0.5 |
| JN10 | 0.14 | 0.21 | NA | 0.5 |
| JN11 | 0.25 | 0.36 | NA | 0.5 |
| JN12 | 0.09 | 0.10 | NA | 0.5 |
| JN13 | 0.14 | 0.18 | NA | 4 |
| JN14 | 0.15 | 0.03 | NA | 4 |
| JN15 | 0.62 | 0.71 | NA | 4.75 |
| JN16 | 0.63 | 0.07 | 0.30 | 1.4/2.33* |

* The first number is the years between the first and second timepoints while the second number is the years between the second and third timepoint. NA, not available.

**Table S3. Determination of HBV evolutionary rate.**

|  | | Relaxed lognormal clock model | | |  | Strict lognormal clock model | | |
| --- | --- | --- | --- | --- | --- | --- | --- | --- |
|  |  | Constant coalescent | Exponential coalescent | Bayesian skyline |  | Constant coalescent | Exponential coalescent | Bayesian skyline |
| JN07 |  | 6.937 × 10^-4^ | 2.252 × 10^-4^ | 5.445 × 10^-4^ |  | 4.255 × 10^-4^ | 1.384 × 10^-4^ | 1.428 × 10^-4^ |
| JN08 |  | 4.928 × 10^-4^ | 6.304 × 10^-5^ | 8.234 × 10^-4^ |  | 3.727 × 10^-4^ | 5.191 × 10^-4^ | 1.981 × 10^-4^ |
| JN09 |  | 5.581 × 10^-4^ | 4.784 × 10^-5^ | 2.119 × 10^-4^ |  | 4.425 × 10^-4^ | 7.729 × 10^-5^ | 2.868 × 10^-5^ |
| JN10 |  | 8.664 × 10^-4^ | 2.311 × 10^-4^ | 8.015 × 10^-4^ |  | 6.614 × 10^-4^ | 2.115 × 10^-4^ | 6.272 × 10^-4^ |
| JN12 |  | 5.019 × 10^-4^ | 6.561 × 10^-5^ | 2.581× 10^-4^ |  | 5.856 × 10^-4^ | 9.461 × 10^-5^ | 1.064 × 10^-4^ |
| JN13 |  | 1.402 × 10^-4^ | 6.974 × 10^-5^ | 5.019 × 10^-5^ |  | 1.148 × 10^-4^ | 8.318 × 10^-5^ | 7.121 × 10^-5^ |
| JN15 |  | 4.724 × 10^-4^ | 4.347 × 10^-4^ | 4.576 × 10^-4^ |  | 4.097 × 10^-4^ | 3.710 × 10^-4^ | 3.992 × 10^-4^ |
| JN14 |  | 2.209 × 10^-3^ | 3.127 × 10^-3^ | 1.091 × 10^-3^ |  | 8.825 × 10^-4^ | 1.045 × 10^-3^ | 8.379 × 10^-4^ |
| JN16* | T1-T2 | 4.724 × 10^-3^ | 1.139 × 10^-2^ | 3.025 × 10^-3^ |  | 2.314 × 10^-3^ | 4.538 × 10^-3^ | 2.073 × 10^-4^ |
|  | T2-T3 | 8.662 × 10^-4^ | 8.560 × 10^-4^ | 9.168 × 10^-4^ |  | 8.217 × 10^-4^ | 8.318 × 10^-4^ | 8.862 × 10^-4^ |
| JN11 |  | 1.508 × 10^-3^ | 1.746 × 10^-3^ | 1.808 × 10^-3^ |  | 1.366 × 10^-3^ | 1.429 × 10^-3^ | - 1. 10^-3^ |

Evolutionary rates are expressed as mean substitutions per site per year.

^*^ Evolutionary rates were determined between the first timepoint (T1) and the second timepoint (T2) as well as between the second timepoint (T2) and the third timepoint(T3).

The rates in blue represent the best fit model for estimating the evolutionary rates as determined by path sampling and are used for Table 2.

**Figure legends**

**Fig S1. Maximum likelihood phylogenetic analysis and Genetic analysis of the near full-length HBV genome sequences from the CHB patients.** A total of 780 near full-length HBV genome sequences from 16 CHB patients were analyzed together with the HBV genotypes B and C reference sequences. The maximum likelihood phylogenetic tree was constructed using PhyML 3.0 with the GTR model. The branch support values were assessed using Approximate Likelihood Ratio Test (aLRT). Sequences from the first, second and third timepoints are indicated by red, blue, and green dots, respectively. The reference sequences are shown in black. The aLRT values exceeding 70% are shown for the nodes.

**Fig S2. Genetic analysis of near full-length HBV genome sequences from the CHB patients.** Phylogenetic trees and highlighter plots of the multiple near full-length HBV genome sequences obtained by SGS from each CHB patient. The phylogenetic tree was constructed by the maximum likelihood method using the GTR model and its reliability was estimated by 1,000 bootstrap replicates. Each tree was rooted at the consensus sequence (black square). Each line in the highlighter plot represents a near full-length genome sequence and the colored marks on the line represent nucleotide differences compared to the consensus sequence (top line). The positions of the substitutions in the HBV genome are shown on the bottom.

**Fig S3. Genetic evolution between two different timepoints in the CHB patients.** The multiple near full-length HBV genome sequences obtained by SGS at two timepoints from each CHB patient were analyzed. Each highlighter plot shows the positions of all mutations in each HBV genome sequence, compared to the consensus sequence of the all sequence obtained at the first timepoint (top thick line). Each line represents a near full-length HBV genome sequence. Mutations and gaps are color-coded and their locations in the viral genome are shown at the bottom. The phylogenetic trees were constructed by the maximum likelihood method with the GTR model. Its reliability was estimated by 1,000 bootstrap replicates. The colors of dots represent viral sequences from different timepoints.

**Fig S4. The role of recombination in the hard selective sweep.** Maximum likelihood trees and highlighter plots were generated with the near full-length genome **(A)**, 5’-half genome **(B)** and 3’-half genome **(C)** sequences from JN14. Each highlighter plot shows the positions of all mutations in each HBV genome sequence, compared to one of the majority sequences (top line). Each line represents an HBV genome sequence. Mutations and gaps are color-coded and their locations in the viral genome are shown at the bottom. The black triangles indicate the preexisting mutations that were selected by the hard selective sweep. The phylogenetic trees were constructed by the maximum likelihood method with the GTR model. Its reliability was estimated by 1,000 bootstrap replicates. The colors of dots represent viral sequences from different timepoints. **(D)** The two recombination regions (5’-half genome in yellow and 3’-half genomes in red) and breakpoint (an arrow at position 1321) are schematically presented.

**Fig S5. Severe reduction of both synonymous and nonsynonymous mutations during the hard selective sweep.** Cumulative plots of each codon average behavior for all sequences compared for the *PreC_Core*, *PreS1_S2_S* and *X* genes at different timepoints for synonymous mutations (green), non-synonymous (red) mutations and indels (blue). Values of ω denote average ratios of the rate of non-synonymous substitutions per non-synonymous site (dN/dS) for each sample. The black arrow indicates the site which was under significant selection determined by MEME analysis (p = 0.04).

**Fig S6. Analysis of the accumulation of near full-length genome mutations in viruses from patients without HSS.** Cumulative plots of each codon average behavior for all sequences compared for the *Pol*, *PreC_Core*, *PreS1_S2_S* and *X* genes at different timepoints for synonymous mutations (green), non-synonymous (red) mutations and indels (blue). Values of ω denote average ratios of the rate of non-synonymous substitutions per non-synonymous site (dN/dS) for each sample.

**Fig S7. The predominant and fixed mutations are selected by the hard selective sweep.** Synonymous (green ticks) and non-synonymous (red ticks) mutations in four ORFs in the HBV genome before and after HSS in JN14, JN16, and JN11 **(A)** and between the second and third timepoints in JN16 **(B)** are shown in the Highlighter plots. All sequences from the same patient were compared to the consensus sequence (top thick line) of the sequences from the first timepoint. The amino acid substitutions in known HLA-I and HLA-II restricted epitopes are indicated by red and blue triangles, respectively. The two wild type amino acid toggle sites (F and Y at position 335; I and M at position 387) are marked with asterisks. The overlapping ORFs in the *Pol* gene are indicated with color lines. The *S* gene and the major hydrophilic region in the *PreS1_S2_S* gene are indicated with blue and orange lines, respectively.

**Fig S8. Shannon entropy analysis of nearly full-length HBV sequences from 10 CHB patients at different time points.** The x-axis represents the nucleotide position, the upper half of the y-axis represents the entropy value of the viral quasispecies at the first time point, and the lower half represents the entropy value at the second timepoint.
